# Evaluation of EGFR-TKIs and ICIs treatment stratification in non-small cell lung cancer using an encrypted multidimensional radiomics approach

**DOI:** 10.1186/s40644-025-00824-w

**Published:** 2025-01-20

**Authors:** Xingping Zhang, Xingting Qiu, Yue Zhang, Qingwen Lai, Yanchun Zhang, Guijuan Zhang

**Affiliations:** 1 School of Medical Information Engineering, Gannan Medical University, Ganzhou, 341000 China; 2 Department of Respiratory and Critical Care, First Affiliated Hospital of Gannan Medical University, Ganzhou, 341000 China; 3 Institute for Sustainable Industries and Liveable Cities, Victoria University, Melbourne, 3011 Australia; 4 School of Computer Science and Technology, Zhejiang Normal University, Jinhua, 321000 China; 5 Department of Radiology, First Affiliated Hospital of Gannan Medical University, Ganzhou, 341000 China; 6 Department of New Networks, Peng Cheng Laboratory, Shenzhen, 518000 China

**Keywords:** Radiomics imaging biomarkers, EGFR-TKIs therapy, ICIs therapy, Privacy protection, Non-small cell lung cancer

## Abstract

**Background:**

Radiomics holds great potential for the noninvasive evaluation of EGFR-TKIs and ICIs responses, but data privacy and model robustness challenges limit its current efficacy and safety. This study aims to develop and validate an encrypted multidimensional radiomics approach to enhance the stratification and analysis of therapeutic responses.

**Materials and methods:**

This multicenter study incorporated various data types from 506 NSCLC patients, which underwent preprocessing through anonymization methods and were securely encrypted using the AES-CBC algorithm. We developed one clinical model and three radiomics models based on clinical factors and radiomics scores (RadScore) of three distinct regions to evaluate treatment response. Additionally, an integrated radiomics-clinical model was created by combining clinical factors with RadScore. The study also explored the association between different EGFR mutations and PD-1/PD-L1 expression in radiomics biomarkers.

**Findings:**

The radiomics-clinical model demonstrated high performance, with AUC values as follows: EGFR (0.884), 19Del (0.894), L858R (0.881), T790M (0.900), and PD-1/PD-L1 expression (0.893) in the test set. This model outperformed both clinical and single radiomics models. Decision curve analysis further supported its superior clinical utility. Additionally, our findings suggest that the efficacy of EGFR-TKIs and ICIs therapy may not depend on detecting a singular tumor feature or cell type.

**Conclusion:**

The proposed method effectively balances the level of evidence with privacy protection, enhancing the study’s validity and security. Therefore, radiomics biomarkers are expected to complement molecular biology analyses and guide therapeutic strategies for EGFR-TKIs, ICIs, and their combinations.

**Supplementary Information:**

The online version contains supplementary material available at 10.1186/s40644-025-00824-w.

## Introduction

In recent years, advancements in molecular biology have brought about a significant transformation in the landscape of non-small cell lung cancer (NSCLC) treatment. Targeted therapies, particularly those involving epidermal growth factor receptor (EGFR) mutation, have proven to be instrumental in prolonging the survival of affected patients [1, 2]. The predominant activating mutations, exon 19 deletion (19Del) and exon 21 L858R missense, encompass approximately 90% of EGFR mutations in NSCLC [3]. Patients carrying these mutations exhibit a favorable response to EGFR tyrosine kinase inhibitors (EGFR-TKIs), but other mutations may be insensitive [4, 5]. Despite initial positive outcomes, most patients develop resistance to first- or second-generation EGFR-TKIs within 8 to 13 months, resulting in a poorer prognosis [6, 7]. Notably, the EGFR-T790M mutation accounts for 50–60% of cases involving acquired resistance mechanisms.

Immunotherapy is another novel treatment modality that inhibits immune checkpoints, preventing tumor cells from escaping immune surveillance [2, 8]. Immune checkpoint inhibitors (ICIs) targeting anti-programmed cell death protein 1 (PD-1) and anti-programmed cell death ligand 1 (PD-L1) are spearheading a revolution in immunotherapy. Numerous studies have demonstrated that this therapy can significantly improve long-term survival in NSCLC patients [3, 8], particularly those without actionable mutations. However, only about 20% of patients respond favorably to this treatment. Furthermore, the optimal therapeutic strategy remains unclear after resistance to EGFR-TKIs. Some studies suggest that EGFR-TKIs therapy affects PD-L1 production [9, 10], and immunotherapy may offer a new option for resistant patients. Therefore, accurately identifying patients responsive to EGFR-TKIs and ICIs therapies is essential for optimizing and personalizing treatment regimens.

In recent years, radiomics has gained widespread attention as a noninvasive tumor analysis tool due to its ability to extract many quantitative features from medical images in a high-throughput manner [11]. These features reflect the phenotypic characteristics and heterogeneity of tumors and provide critical information regarding tumor sensitivity to treatment, thus supporting clinical diagnosis and treatment response prediction [12–14]. Despite its great potential, the development of radiomics technology is still limited by model robustness and scalability issues [15]. For instance, variations in image acquisition, reconstruction protocols, and preprocessing procedures can affect the reliability of the results. Additionally, it has been shown that features from the largest 2D region of the lesion perform comparably to radiomic features from a 3D region of interest (ROI) in terms of predictive performance [16, 17]. Meanwhile, peri-tumor region features can significantly improve prediction accuracy in the medical field due to their unique ability to reflect both the tumor and its microenvironment [18–20].

On the other hand, an often-overlooked issue is that medical images contain sensitive information related to patient identity and health status. During storage and sharing, this information is vulnerable to unauthorized processing and privacy breaches [21, 22], which limits the openness and clinical applicability of the research. Currently, most studies have focused on medical image encryption techniques [21], but no reports have been on combining image encryption with radiomics. Therefore, integrating multi-region features into radiomic analysis can optimize traditional methods while combining privacy-preserving data encryption mechanisms, which can further improve the applicability of radiomics in clinical settings. This combined strategy is expected to yield benefits in constructing more accurate and robust predictive models.

In existing studies, radiomic features extracted from CT images of primary tumors have been used to predict 19Del, L858R, and T790M mutations [23, 24], as well as PD-1/PD-L1 expression [25] in NSCLC. However, these studies have focused only on regional features of primary tumors, ignoring peritumoral regions or potentially complementary bi-regional features. Additionally, most studies are limited to predicting a single or a few mutation types. Combining multiregional characterization could help more comprehensively assess patient responses to EGFR-TKIs, ICIs, and their potential combination therapies.

In this study, we combined image encryption with radiomics to develop a noninvasive preoperative model for predicting the response to treatment with EGFR-TKIs and ICIs based on radiomic features from three tumor regions and clinical factors. Additionally, we explored potential relationships between responses to different therapies through a stratified analysis of radiomics imaging biomarkers.

## Materials and methods

### Patient population and study design

The study population was retrospectively selected from two sources: the First Affiliated Hospital of Gannan Medical University (FAHGMU) in China and the publicly accessible “NSCLC Radiogenomics” dataset from The Cancer Imaging Archive (TCIA) in the United States. A total of 368 NSCLC patients meeting the inclusion criteria were enrolled in the FAHGMU cohort, with recruitment conducted in two phases: from August 2017 to April 2021 (FAHGMU1) and from May 2021 to December 2021 (FAHGMU2). Detailed inclusion and exclusion criteria are provided in Appendix A.

The samples in the TCIA cohort were obtained from two institutions in the United States. They included clinical data, CT scans, EGFR mutation status, and RNA sequencing (RNA-seq) data. The screening criteria for the TCIA cohort were identical to those used for the FAHGMU cohort, and data from 138 patients who met the quality standards were ultimately included. Cases from the FAHGMU1 cohort were designated as the training set (Cohort I). Based on previous work and pre-experimental results, the model was trained and optimized using a five-fold cross-validation method with 30 repetitions. The combined cases from the FAHGMU2 and TCIA cohorts served as an independent external test set (designated as Cohort II).

**Fig. 1:**
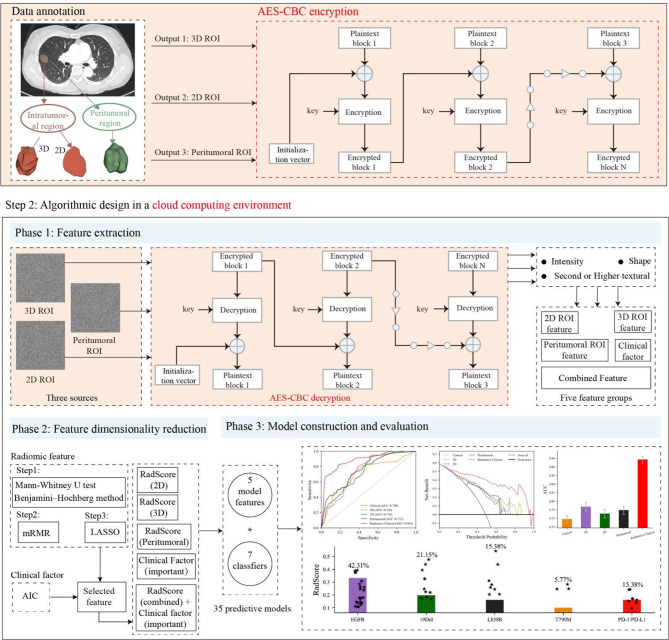
Study design for this integrative radiomics-clinical approach

As depicted in Fig. 1, the research design consists of two primary steps. Step 1 is conducted within a secure local environment. Firstly, the CT images undergo preprocessing to identify two distinct intratumoral regions and one peritumoral region. Subsequently, these delineated regions are encrypted using an encryption algorithm to ensure data security for the subsequent step.

Step 2 is executed within a cloud computing environment and encompasses three stages. First, radiomic features are extracted from the three encrypted ROIs using a decryption algorithm. Second, a sequential three-step procedure is applied to identify the most stable and discriminative features. Third, the selected features were integrated with clinical factors and analyzed using seven classifiers to develop a baseline clinical model, three independent radiomics models, and a comprehensive radiomics-clinical model. Finally, the performance of all models is comprehensively evaluated and compared.

### Data preprocessing

Regarding data definition, based on genetic test reports, EGFR mutation subtypes were categorized as 19Del mutation, L858R mutation, T790M resistance mutation, and EGFR wild-type. The definition of PD-1/PD-L1 expression status is derived from the tumor proportion score (TPS) and categorized into two groups: expressed and non-expressed. More detailed definitions and classifications are provided in Appendix B.

For CT imaging, all patients underwent a chest CT scan; however, the scanning protocols and acquisition parameters varied. FAHGMU used two scanners to acquire the CT images, and the detailed scanning protocols and acquisition parameters are provided in Appendix C. For TCIA patients, although specific imaging details were not available, planar resolution and slice thickness could be extrapolated from the available image data.

Two medical experts with 5 and 10 years of experience manually segmented and corrected the 3D ROI for tumor segmentation. Subsequently, an internal algorithm automatically selects the cross-sectional slice with the largest area from the 3D ROI as the 2D ROI. It extends the boundary outward by 3 mm to generate the peri-tumor ROI. Figure 1 (Step 1) illustrates the three ROIs for one of the patients. The detailed segmentation process is outlined in Appendix D.

Finally, the voxel spacing of the 2D ROI, 3D ROI, and peritumoral ROI was resampled to 0.8 mm $\:\times\:$ 0.8 mm, 1.0 mm $\:\times\:$ 1.0 mm $\:\times\:$ 1.0 mm, and 1.0 mm $\:\times\:$ 1.0 mm $\:\times\:$ 1.0 mm, respectively, based on the resolution and fidelity distributions of the CT images. For more details on the resampling analysis and the design process, please refer to Appendix E.

### Data anonymization and image encryption

Regarding data anonymization, only the relevant clinical factors and indicators are retained through file parsing and processing of textual data. For CT image encryption, the original images are transformed into encrypted versions by modifying pixel data, rendering them noisy or unintelligible, thereby protecting sensitive information. This study utilizes the advanced encryption standard (AES) algorithm to ensure the reversibility of the encryption process while maintaining image quality. A detailed theoretical explanation of this algorithm is presented in Appendix E.

In the encryption process illustrated in Fig. 1 (Step 1), the cipher-block chaining (CBC) mode necessitates the generation of a random initialization vector (IV) as an initial step. This IV is then XOR-ed with the first plaintext block. Once the first encrypted block is produced, subsequent encrypted blocks are derived by XOR-ing the preceding encrypted block with the current plaintext block.

As illustrated in Fig. 1 (Step 2), the decryption process in CBC mode closely resembles the encryption procedure. First, the initial ciphertext block is decrypted using the key, and the resulting output is XOR-ed with the IV to produce the first plaintext block. For subsequent plaintext blocks, the process involves XOR-ing the previous ciphertext block with the decryption result of the current ciphertext block.

### Radiomic feature extraction

Before proceeding with feature extraction, the encrypted ROI must be decrypted using the AES-CBC decryption program. The resulting decrypted data will serve as input for the feature extraction process; however, it will not be retained. Subsequently, the voxel spacing for the three ROI types has been standardized to 0.8 mm^2^ for 2D ROIs and 1.0 mm^3^ for 3D and peritumoral ROIs. This standardization aims to maximize the advantages of each ROI type. For a detailed analysis of resampling and the trade-offs associated with different voxel spacing sizes, please refer to Appendix F.

We systematically characterized the radiological phenotype of each tumor region by two distinct feature categories: raw features and wavelet features. The raw feature ensemble comprised 14 shape features, 19 first-order statistical features, 24 GLCM features, 16 GLRLM features, 16 GLSZM features, 5 NGTDM features, and 14 GLDM features. Concerning the wavelet feature sets, we extracted original feature sets from each of the eight derived images, excluding shape features. Subsequently, a comprehensive total of 743 radiomic features were extracted for each ROI. The definition of these features followed the guidelines set by the Image Biomarker Standardization Initiative. Further details on the feature extraction can be found in previous works [26] and the corresponding parameter settings provided in Table S1.

### Selection of optimal features

Before proceeding with subsequent analyses, the training and test sets underwent a standardization process using Z-score normalization. This process involved parameters derived from the mean and standard deviation calculated from the training set. Following normalization, we implemented a careful three-step feature selection procedure. Initially, discriminative features were selectively retained based on the Mann-Whitney U test, with correction facilitated through the false discovery rate (FDR = 5%) Benjamini-Hochberg procedure (p-value < 0.05). Subsequently, the minimum Redundancy Maximum Relevance algorithm was applied to eliminate redundant and extraneous features. Lastly, LASSO regression was employed to identify optimal descriptors with non-zero coefficients. The LASSO hyperparameter (lambda) was determined through 5-fold cross-validation, optimizing against the overall misclassification error (MCE). Post-selection, the radiomics scores (RadScore) formulation occurred as a linear amalgamation of the chosen superior features, each weighted according to their respective coefficients as ascertained in the training set.

### Model building and performance evaluation

The selected optimal feature set served as the foundation for a combinatorial strategy that utilized seven commonly employed machine learning classifiers, aiming to discern suitable predictive models. The optimization parameters of these algorithms were determined through a 5-fold cross-validated grid search method. We developed specific models using the RadScore of the three tumor regions, including a 2D radiomics model, a 3D radiomics model, and a peritumoral radiomics model, respectively. Simultaneously, we established a quantitative clinical model using relevant clinical features. Subsequently, the Akaike Information Criterion method was applied to identify the most important clinical factors. These factors were then integrated with the RadScore derived from the combined feature set, constructing a comprehensive radiomics-clinical model. A detailed list of the employed classifiers is provided in Appendix G.

To assess the predictive capabilities for responses to EGFR-TKIs and ICIs therapies, models derived from the training set underwent validation in an independent test set. We employed a 30-times 5-fold stratified cross-validation method to address categorical sample size limitations during model training and optimization. This approach aimed to provide an unbiased estimation of the models, mitigating overfitting and enhancing result robustness. Model performance was evaluated through the area under the receiver operating characteristic (ROC) curve, with higher AUC values indicating superior discrimination by the corresponding model features. Decision curve analysis (DCA) assessed the clinical utility of the clinical, 2D, 3D, peritumoral, and radiomics-clinical models. The DeLong test was utilized to discern differences in AUC among all models. Ultimately, these developed models were comprehensively compared and analyzed in an independent test set to ensure their efficacy.

### Stratified analysis of radiomics imaging biomarkers

To identify disparities in the distribution of radiomics imaging biomarkers between therapies involving EGFR-TKIs and ICIs, we investigated to assess the consistency of RadScore values across distinct EGFR mutations (specifically, EGFR+, 19Del, L858R, and T790M) and PD-1/PD-L1 expression levels. Subsequent subgroup analyses, based on RadScore distributions, were performed to elucidate the associations between radiomics imaging biomarkers and the therapeutic response to EGFR-TKIs and ICIs. Critical thresholds for different EGFR mutation subtypes and PD-1/PD-L1 expression were established by referring to the quartiles of RadScore within the training set. Patient stratification followed, categorizing individuals into three groups (RadScore-high, RadScore-median, and RadScore-low) based on tertiles, with detailed delineation of group boundaries in Appendix H.

To elucidate the complex relationship between EGFR-TKIs and ICIs, particularly in the context of ICIs administered after resistance to EGFR-TKIs, we conducted a comprehensive analysis of correlation coefficients for radiomics imaging biomarkers across different treatment modalities. The primary metric employed to define therapies involving EGFR-TKIs and ICIs is RadScore’s Spearman’s rho, which evaluates the correlation between EGFR mutation status (including EGFR wild type, 19Del mutation, and L858R mutation) and PD-1/PD-L1 expression. Additionally, RadScore’s Spearman’s rho for the T790M mutation about PD-1/PD-L1 expression serves as a reference for assessing combination therapies. Subsequently, correlation heatmaps were generated and subjected to a meticulous comparative analysis across these varied scenarios.

### Statistical methods

Radiomic analyses were conducted using an internally developed Python program (version 3.7.6). Continuous variables and RadScore were assessed through the Wilcoxon signed-rank test, while categorical variables underwent Fisher’s exact test. To address multiple comparisons, p-values were adjusted using a false discovery rate procedure, following the Benjamini-Hochberg method with a 5% threshold. RNA-seq data were quantified in Cohort II using the reads per kilobase million method. Model performance evaluation included ROC curves, AUC, accuracy, sensitivity, and specificity, with 95% confidence intervals computed through 1000 bootstrap samples. Spearman’s correlation elucidated the relationship between RadScore and responses to EGFR-TKIs and ICIs therapies. The pre-determined significance level was set at a p-value < 0.05.

## Results

### Statistical characteristics of patients

The detailed clinical information and RadScore for all patients are presented in Table 1. Statistical analysis of the combined datasets (Cohort I and Cohort II) revealed that the prevalence of EGFR mutation, 19Del mutation, and L858R mutation, which are associated with EGFR-TKIs therapies, were 38.5%, 19.1%, and 14.9%, respectively. The prevalence of the T790M mutation, which confers resistance to EGFR-TKIs, was 9.2%. Additionally, 47.1% of patients with PD-1/PD-L1 expression responded to ICIs therapy. Clinical characteristics in Table 1 with a p-value < 0.05 were considered statistically significant.

For the three RadScore calculated from the 2D, 3D, and peritumoral regions, the mean RadScore for positive patients in the EGFR, 19Del, L858R, T790M, and PD-1/PD-L1 groups were higher than those for negative patients. A significant difference was observed in all cases (p-value < 0.001), except for the 2D RadScore (p-value = 0.015) and the peritumoral RadScore (p-value = 0.012) in the T790M group. Thus, RadScore is an independent predictor of response to EGFR-TKIs and ICIs therapies.

**Table 1:** Clinical characteristics and RadScore of all patients

Characteristic	Cohort I (*n* = 303)	Cohort II (*n* = 203)	*P* value	Genotypes	Training (Cohort I)	Test (Cohort II)	*P* value
Age (years)			< 0.001	EGFR			< 0.001
Mean ± SD	62.1 ± 10.8	67.7 ± 9.5		Mutant	137 (45.2)	55 (29.9)	
Gender			0.014	Wildtype	166 (54.8)	129 (70.1)	
Male	190 (62.7)	140 (69.0)		19Del			< 0.001
Female	113 (37.3)	63 (31.0)		Mutant	59 (26.2)	21 (14.0)	
Smoking status			< 0.001	Wildtype	166 (73.8)	129 (86.0)	
Smoker	151 (49.8)	143 (70.4)		L858R			< 0.001
Nonsmoker	152 (50.2)	60 (29.6)		Mutant	44 (5.8)	16 (11.0)	
Tumor location			0.020	Wildtype	166 (79.0)	129 (89.0)	
RUL	89 (29.4)	70 (34.5)		T790M			< 0.001
RML	27 (8.9)	19 (9.4)		Mutant	19 (10.3)	9 (6.5)	
RLL	53 (17.5)	35 (17.2)		Wildtype	166 (89.7)	129 (93.5)	
LUL	92 (30.4)	53 (26.1)		**Immune molecule**	**Training (Cohort I)**	**Test (Cohort II)**	**P value**
LLL	42 (13.9)	26 (12.8)		PD-1/PD- L1			0.016
LVI			< 0.001	Positive	58 (51.3)	26 (40.0)	
Present	120 (39.6)	43 (21.2)		Negative	55 (48.7)	39 (60.0)	
Absent	183 (60.4)	160 (78.8)		**RadScore**	**Training (Cohort I)**	**Test (Cohort II)**	**P value**
PI			< 0.001	2D region Median (six groups)	(0.051, 0.131, 0.0976, 0.074, -0.004)	(-0.027, -0.119, -0.145, -0.046, -0.066)	< 0.001
Yes	232 (76.6)	87 (42.9)					
No	71 (23.4)	116 (57.1)		3D region Median (six groups)	(0.050, 0.101, 0.092, 0.178, -0.018)	(0.000, -0.131, -0.023, -0.094, -0.022)	< 0.001
T stage			< 0.001				
T4	100 (33.0)	25 (12.3)		Peritumoral region Median (six groups)	(-0.006, 0.072, 0.039, 0.138, -0.032)	(-0.051, -0.064, -0.056, -0.067, 0.003)	< 0.001
Other	203 (67.0)	178 (87.7)					

Note Data are presented as n (%) unless otherwise indicated. Six groups represent EGFR, 19Del, L858R, T790M, and PD-1/PD-L1. Abbreviations: SD, standard deviation; RUL, right upper lobe; RML, right middle lobe; RLL, right lower lobe; LUL, left upper lobe; LLL, left lower lobe; LVI, Lymphovascular invasion; PI, Pleural invasion

### Image decryption and pixel correlation

**Fig. 2:**
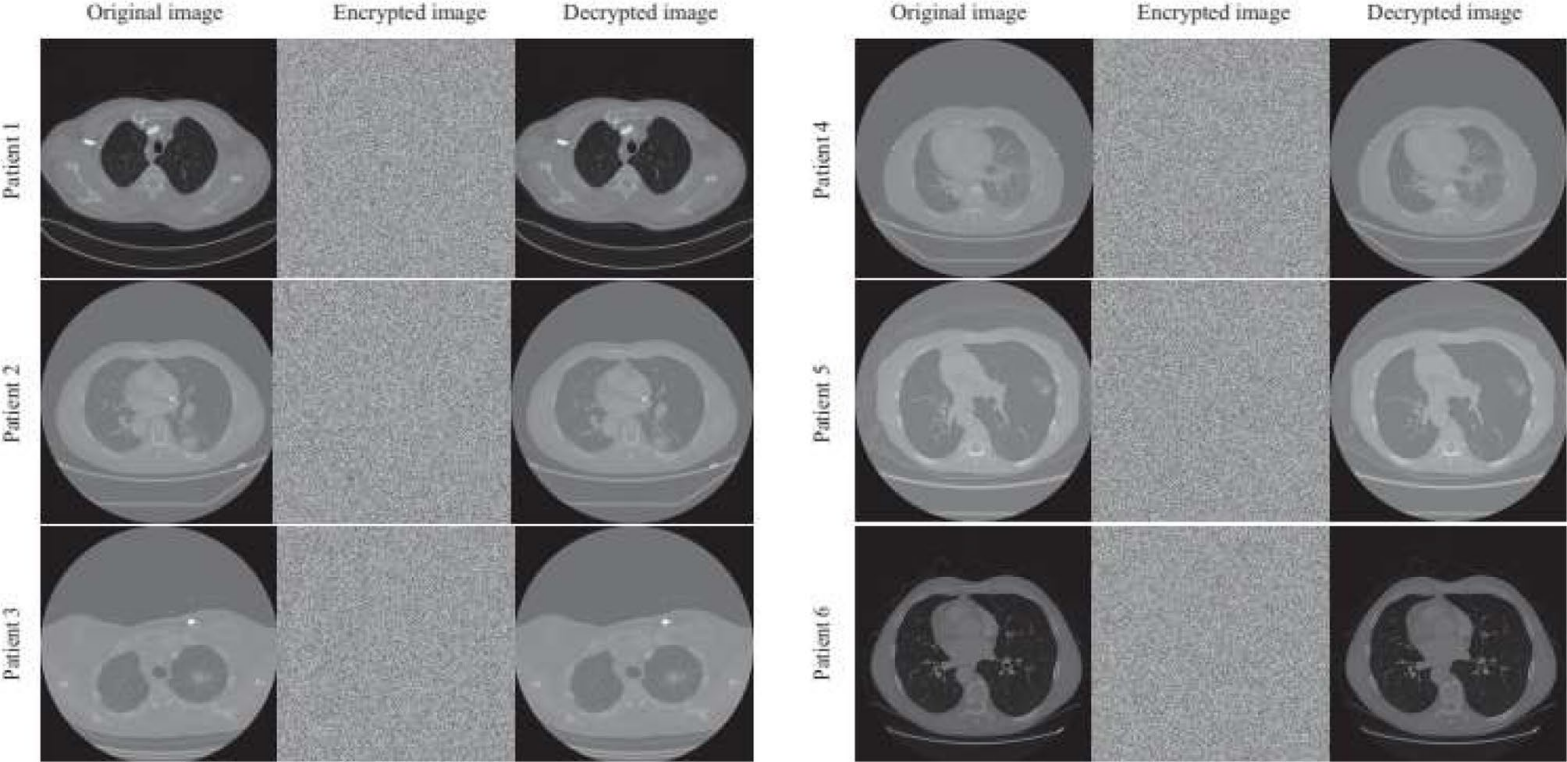
Effect of the AES-CBC encryption algorithm on CT cross-sectional Images of six random patients

Medical diagnosis is highly sensitive to even minor variations in image pixels, making it essential to preserve the quality of encrypted images to prevent any adverse effects on radiomic analysis. The Structural Similarity (SSIM) index is an effective metric for evaluating how brightness, contrast, and structural differences impact the visual quality of an image. The results demonstrate that the SSIM value between each patient’s original CT image and its corresponding decrypted image is 1, confirming that the proposed AES-CBC encryption and decryption process is entirely lossless. As shown in Fig. 2, the encrypted image resembles noise or a meaningless pattern, while the decryption process restores the original image without any data loss.

Pixel correlation is an important indicator of image randomness. In chest CT images, adjacent pixels typically exhibit high correlation; thus, an effective encryption method must significantly reduce this correlation to enhance security. As shown in Table S7, pixel correlation in the encrypted image is substantially reduced in the horizontal, vertical, and diagonal directions, approaching zero. Therefore, the proposed AES-CBC algorithm effectively disrupts pixel correlation, offering strong resistance to statistical attacks.

### Performance in identifying response to EGFR-TKIs therapies

Appendix I provides a detailed description of the predictive model developed for each case, the key features selected, and the formula used to calculate RadScore.

The model’s performance was assessed using independent test data unless otherwise specified. Among EGFR mutation, the AUC values were 0.730 (95% CI, 0.720–0.740), 0.746 (95% CI, 0.737–0.755), and 0.752 (95% CI, 0.742–0.761) for the 2D, 3D, and peritumoral models, respectively. The AUC of the model constructed from clinical factors was 0.708 (95% CI, 0.697–0.718), indicating inferior performance compared to radiomics models based on a single region. However, the radiomics-clinical model exhibited superior performance with an AUC of 0.884 (95% CI, 0.877–0.890). These results suggest that integrating clinical factors, 2D regions, 3D regions, and peritumoral regions offers a valuable and synergistic advantage.

**Fig. 3:**
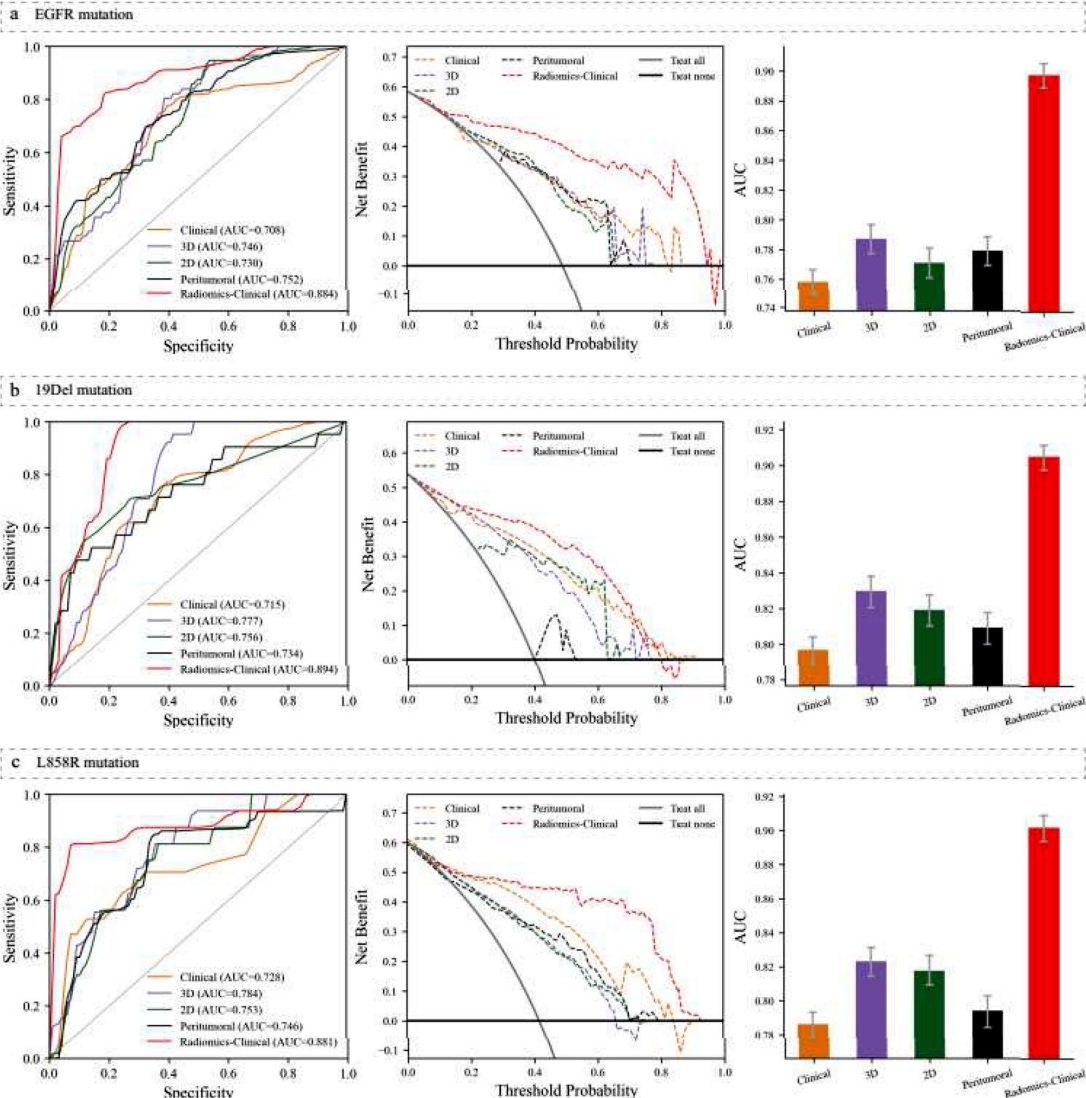
ROC curves (left inset) and DCA curves (middle inset) for each model predicting EGFR mutation (**a**), 19Del mutation (**b**), and L858R mutation (**c**) in the independent test set, and AUC distribution (right inset) in the training set

The ROC curves, DCA, and AUC distributions for each model are depicted in the three insets of Fig. 3a. The DCA reveals that the radiomics-clinical model (in red) encompasses a larger area than the clinical, 2D, 3D, and peritumoral models. This result suggests that the radiomics-clinical model holds greater promise for clinical decision utility.

In other EGFR mutation groups, similar outcomes were observed. Initially, single region-based radiomics models exhibited superior predictive efficacy compared to the clinical model. The AUC values for predicting the 19Del, L858R, and T790M mutations were as follows: 0.715 (95% CI, 0.701–0.728), 0.728 (95% CI, 0.713–0.745), and 0.746 (95% CI, 0.719–0.774) for the clinical model; 0.756 (95% CI, 0.741–0.772), 0.753 (95% CI, 0.739–0.767), and 0.769 (95% CI, 0.750–0.786) for the 2D model; 0.777 (95% CI, 0.767–0.787), 0.784 (95% CI, 0.772–0.797), and 0.816 (95% CI, 0.796–0.835) for the 3D model; and 0.734 (95% CI, 0.718–0.749), 0.746 (95% CI, 0.729–0.763), and 0.771 (95% CI, 0.757–0.784) for the peritumoral model.

**Fig. 4:**
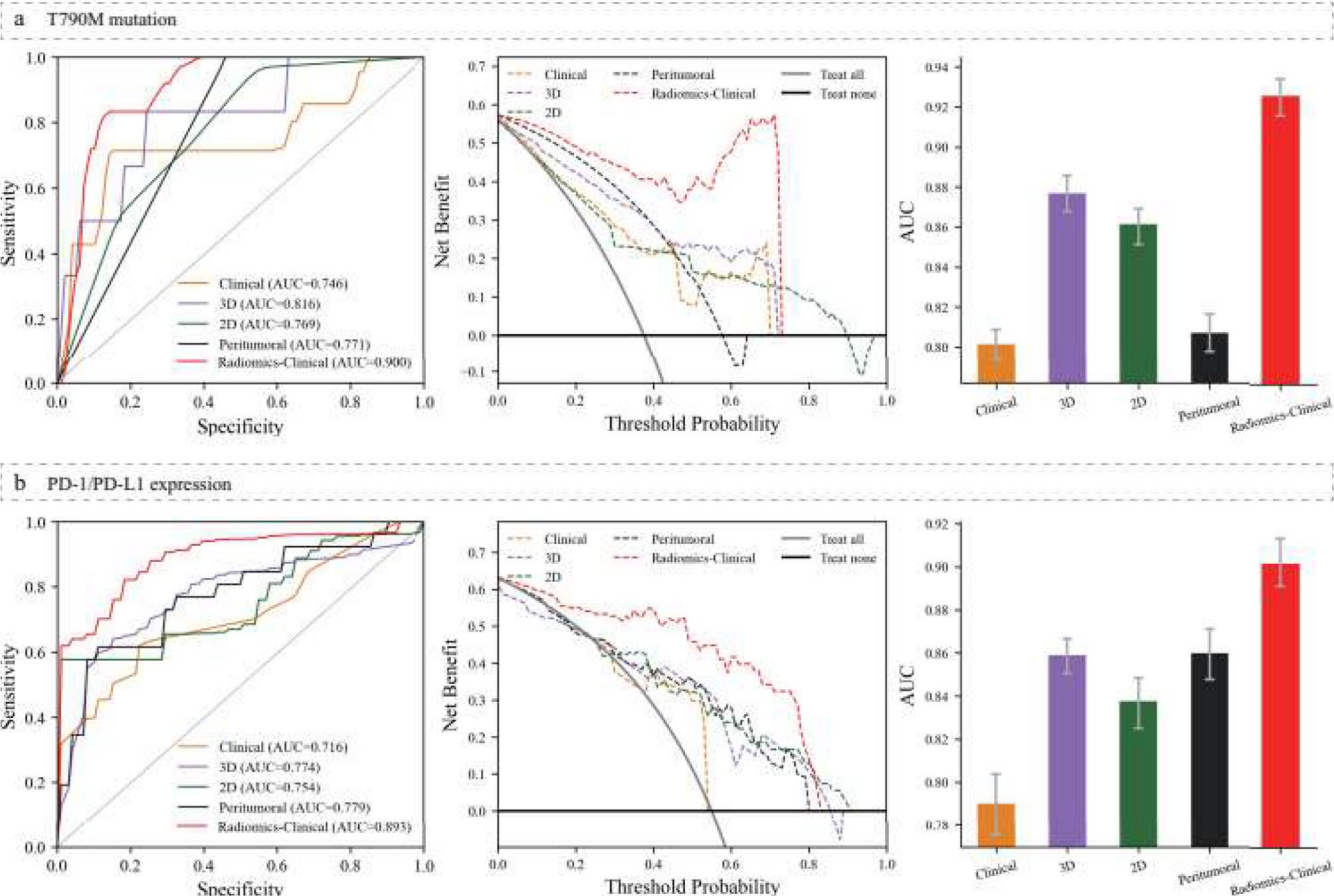
ROC curves (left inset) and DCA curves (middle inset) for each model predicting T790M mutation (**a**) and PD-1/PD-L1 expression (**b**) in the independent test set and AUC distribution (right inset) in the training set

Secondly, our integrated radiomics-clinical model, which combines clinical variables with multiregional RadScore, demonstrated superior performance compared to other models. The model achieved AUCs of 0.894 (95% CI, 0.887–0.901) for the 19Del mutation, 0.881 (95% CI, 0.867–0.896) for the L858R mutation, and 0.900 (95% CI, 0.889–0.910) for the T790M mutation. Figures 3b-c and 4a summarize the performance of each model regarding these EGFR mutations, including ROC curves, DCA curves, and AUC distributions.

The AUCs of the radiomics-clinical models exhibited varying degrees of improvement relative to the conventional single model in predicting the response to EGFR-TKIs therapies. Supplementary performance metrics for each model are delineated in Table S8 for reference.

### Performance in identifying response to ICIs therapies

Concerning identifying patients sensitive to ICI therapies, as depicted in Fig. 4b, the radiomics-clinical model demonstrated the highest performance, with an AUC of 0.893 (95% CI, 0.883–0.903). In comparison, the predictive performance of the 2D, 3D, and peritumoral models was moderate, with AUCs of 0.754 (95% CI, 0.737–0.769), 0.774 (95% CI, 0.758–0.789), and 0.779 (95% CI, 0.764–0.792), respectively. The clinical model exhibited the lowest predictive performance, with an AUC of 0.716 (95% CI, 0.700-0.732).

Figure 4b presents each model’s ROC curve, DCA, and AUC distribution. The integration of key clinical characteristics and RadScore (combined) demonstrated the strongest predictive capability. Additionally, the DCA analysis indicated that the radiomics-clinical model provided a greater net benefit for PD-1/PD-L1 expression than the clinical and single-region radiomics models, enhancing its clinical feasibility.

### Radiomics imaging biomarkers associated with EGFR-TKIs and ICIs therapy responses

This study investigated the association between radiomics imaging biomarkers and therapeutic responses to EGFR-TKIs and ICIs. Notably, the RadScore exhibited significant variations (p-value < 0.001) across different genetic mutations, including the EGFR mutation, 19Del mutation, L858R mutation, T790M mutation, and PD-1/PD-L1 expression. Specifically, the RadScore for the L858R mutation (mean, -0.027; 95% CI, -0.469 to 0.325) and for PD-1/PD-L1 expression (mean, -0.031; 95% CI, -0.293 to 0.169) was significantly lower compared to that for the EGFR mutation (mean, 0.013; 95% CI, -0.273 to 0.334) and the T790M mutation (mean, 0.014; 95% CI, -0.539 to 0.343). Moderate results were observed for the 19Del mutation, with a mean RadScore of 0.008 (95% CI, -0.375 to 0.319).

**Fig. 5:**
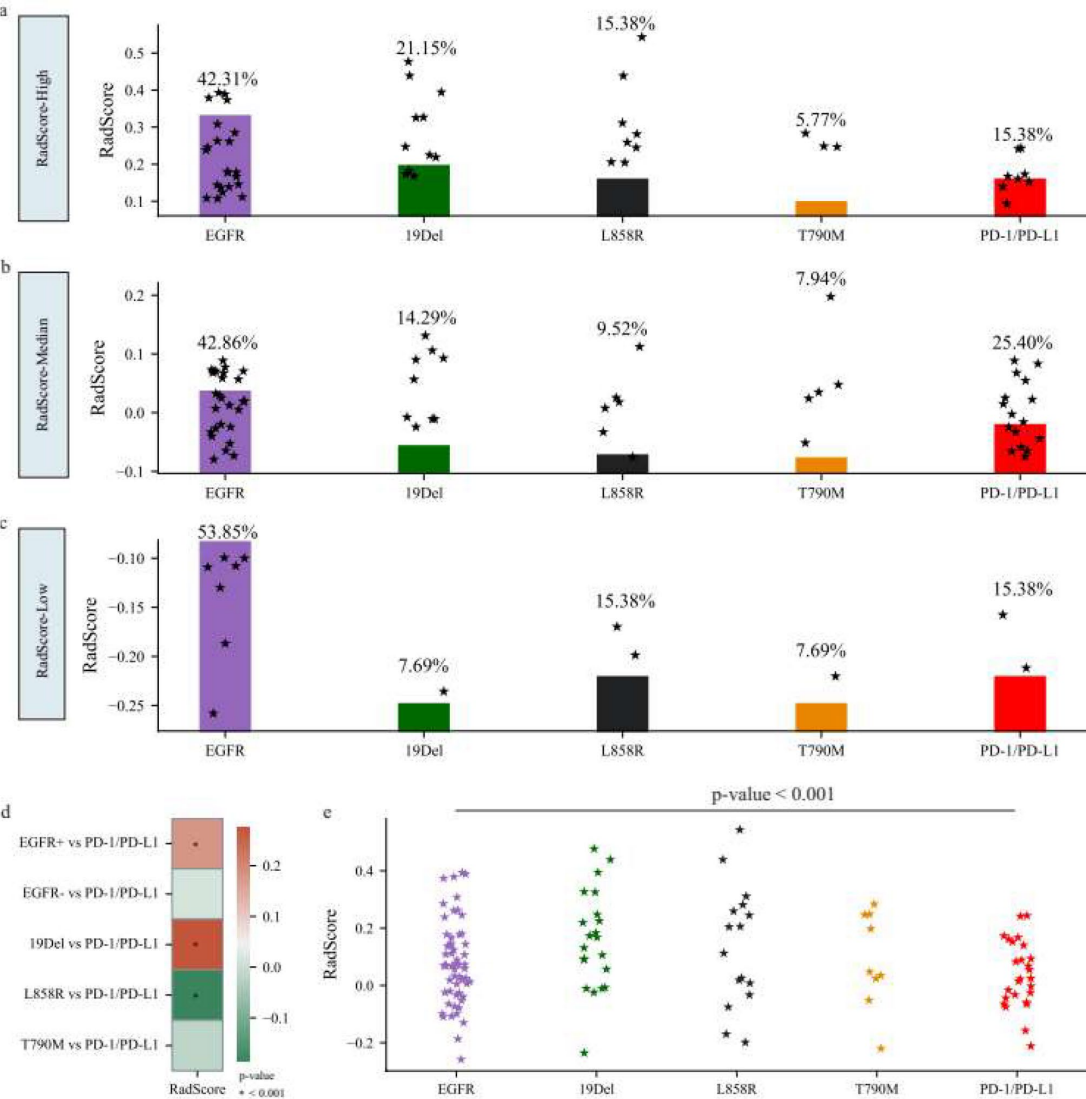
RadScore (combined) analysis of therapeutic responses to EGFR-TKIs and ICIs in independent test set. **a**-**c** RadScore (Combined) for patients in different RadScore groups. **d**, **e** Correlation heatmap and distribution of RadScore (combined) for different therapy responses

When patients were categorized into distinct RadScore groups (see Fig. 5a-c), we observed a significantly higher rate of the 19Del mutation in the RadScore-High group (21.15%) compared to the RadScore-Low group (7.69%). Additionally, the prevalence of patients exhibiting both the L858R mutation and PD-1/PD-L1 expression was 15.38% in the RadScore-High and RadScore-Low groups. In contrast, the rates for the L858R mutation and PD-1/PD-L1 expression in the RadScore-Median group were 9.58% and 25.40%, respectively. Notably, the EGFR mutation rate was the highest, ranging from 42.31 to 53.85%, while the T790M mutation rate was the lowest, ranging from 5.77 to 7.94%.

Given the limited clinical responses to EGFR-TKIs and ICIs, a subgroup analysis was conducted to explore the nuanced relationship between RadScore and therapeutic responses. This investigation aimed to identify associations between specific EGFR mutations and PD-1/PD-L1 expression. As illustrated in Fig. 5d, a statistically significant correlation (p-value < 0.05) emerged among three EGFR mutation statuses: EGFR mutation, 19Del mutation, and L858R mutation.

Specifically, the 19Del mutation exhibited the strongest positive correlation (*r* = 0.274, p-value < 0.001), while the L858R mutation demonstrated a significant negative correlation (*r* = -0.185, p-value < 0.001). The overall EGFR mutation status showed a moderately positive correlation (*r* = 0.178, p-value < 0.01). In contrast, the EGFR wild-type (*r* = 0.012, p-value = 0.065) and T790M mutation (*r* = -0.015, p-value = 0.058) exhibited either a negative correlation or a lack of statistical significance.

In the analysis of RadScore disparities associated with responses to EGFR-TKIs and ICIs, Fig. 5d illustrates the distribution of RadScore across the four EGFR mutation statuses and PD-1/PD-L1 expression levels. The results (see Fig. 5e) reveal a statistically significant variance in RadScore about different therapeutic responses (p-value < 0.001).

**Fig. 6:**
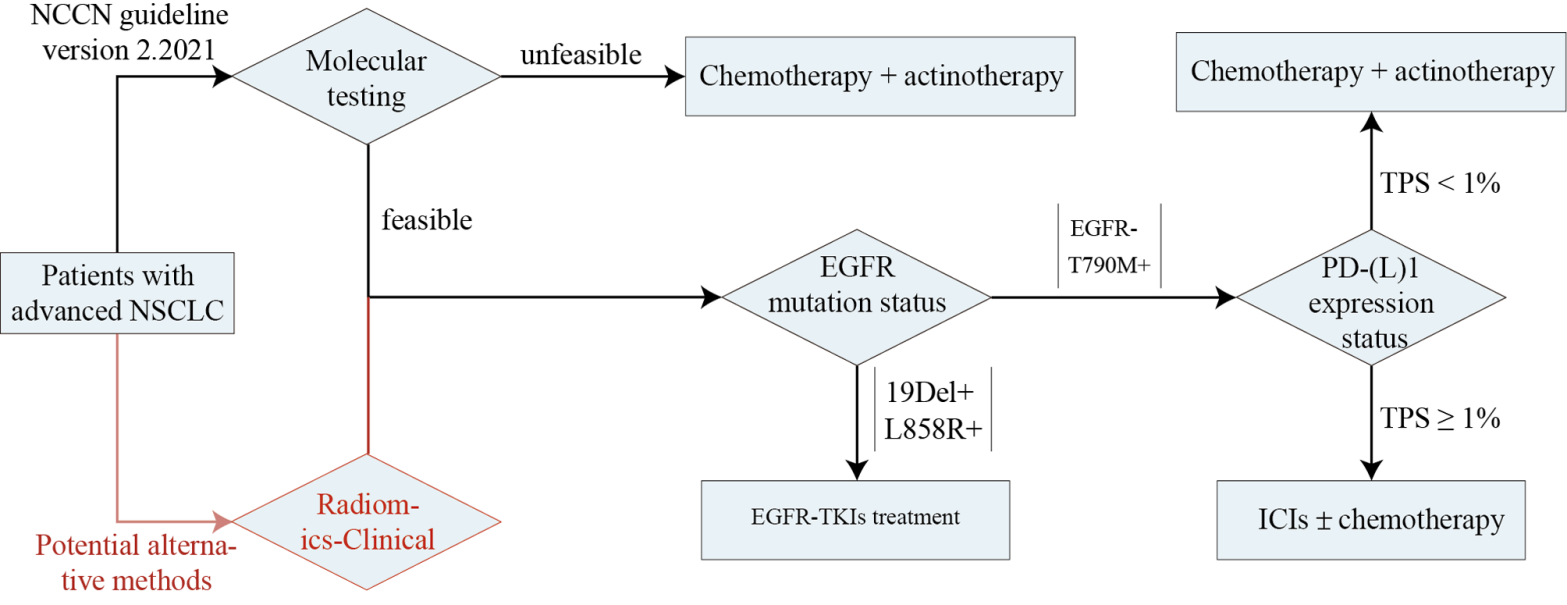
Radiomics biomarkers as potential guides for the treatment of NSCLC with EGFR-TKIs or ICIs. *Abbreviations* NCCN, National Comprehensive Cancer Network

Additionally, Fig. 6 provides an overview of the potential applicability of the proposed radiomics imaging biomarkers in informing treatment strategies for NSCLC, particularly in therapy with EGFR-TKIs or ICIs.

## Discussion

Identifying predictive biomarkers of treatment response to EGFR-TKIs and ICIs is crucial for optimizing treatment decisions, avoiding premature discontinuation of therapy, and preventing the prolongation of ineffective treatment periods. In this study, we extracted radiomic features associated with EGFR mutant subtypes (including the 19Del, L858R, and drug-resistant T790M mutations) and PD-1/PD-L1 expression from CT images. The multi-scale RadScore developed from these features demonstrates significant potential and is easily accessible clinically. More importantly, it strikes a balance between privacy protection and the level of radiographic evidence, which has not been previously reported in the literature. As a multicenter study, we constructed and validated a radiomics-clinical model integrating three tumor regional features with clinical factors using encrypted CT images and clinical data. Furthermore, we explored potential associations between EGFR-TKIs, ICIs therapy, and their combination therapy through a stratified analysis of radiomics imaging biomarkers, offering valuable insights and references for advancing precision medicine.

In recent years, radiomics analysis has been widely used to characterize the biological behavior of malignant tumors, demonstrating significant clinical value. For example, Hu et al. [27] utilized deep learning radio-clinical signatures based on CT to demonstrate excellent performance in predicting the response to neoadjuvant chemotherapy and prognosis in gastric cancer patients, achieving an AUC of 0.82 and a consistency index of 0.64. Lan et al. [28] developed a model combining deep learning features and radiomic features to successfully predict occult lymph node metastasis in patients with early-stage oral and oropharyngeal squamous cell carcinoma. The best AUC obtained in the external validation set was 0.834. Yu et al. [29] pointed out that AI models incorporating multimodal data help reveal the heterogeneity of the tumor microenvironment and significantly enhance risk stratification in breast cancer. These studies suggest that the predictive power of traditional radiomics methods can be further enhanced by incorporating additional effective feature analysis.

Notably, studies have shown that features of the peri-tumor region can reflect subtle changes in the tumor microenvironment and provide additional information about tumor biological heterogeneity [30–32]. However, no study has yet combined features from different regions (particularly peri-tumor regions) to predict the response to EGFR-TKIs or ICIs in lung cancer patients. Radiomics analysis based on multiregional features may offer a new direction for developing more accurate and robust prediction models-an idea further validated in this study.

Specifically, the multiregional radiomics-clinical model proposed in this study outperformed the single-modality feature model in prediction performance. Across all prediction scenarios, the AUCs of the combined model showed improvements of 0.132 to 0.176 (EGFR mutation), 0.117 to 0.179 (19Del mutation), 0.097 to 0.153 (L858R mutation), 0.084 to 0.154 (T790M mutation), and 0.114 to 0.177 (PD-1/PD-L1 expression). These findings suggest combining multiregional features can effectively mine critical information within and around the tumor.

Compared to recent related studies, the present study demonstrated a significant advantage in its predictive ability to distinguish three EGFR mutation subtypes and one immunophenotype. For instance, in predicting the 19Del mutation and L858R mutation, the AUCs reported by Li et al. [33] and Liu et al. [34] using tumor region-based radiomics models were 0.792 and 0.775, and 0.867 and 0.704, respectively. In contrast, the present study achieved AUCs of 0.894 and 0.881. Similarly, for the EGFR-T790M mutation, Fan et al. [35] and Tang et al. [24] reported AUCs of 0.800 and 0.760, respectively, while the present study reached an AUC of 0.900. Moreover, in predicting PD-1/PD-L1 expression, the AUC of the present study was 8.93, surpassing the results of previous studies [36, 37]. These findings highlight the effectiveness and innovation of the multi-region characterization-based approach employed in this study.

Furthermore, this study demonstrated innovation by evaluating treatment responses to EGFR-TKIs and ICIs while ensuring patient data privacy. However, it is important to consider the potential impact of image encryption on radiomics analysis. On the one hand, while the AES algorithm provides lossless encryption, is easily implemented in hardware, and offers high security due to its large key length, striking a balance between security and efficiency remains a challenge. As shown in Table S9, the average time required for decryption and feature extraction per patient ranges from 1.93 to 2.09 s and 10.62 to 14.00 s for 2D, 3D, and peri-tumor regions, respectively. This underscores the need to develop more efficient and lightweight encryption schemes to protect patient privacy and data security better. On the other hand, although this study represents a step forward, its reliance on decrypting encrypted datasets limits the broader applicability of the proposed method. Consequently, conducting radiomics studies in an encrypted environment, without the need to decrypt medical images, may be a promising direction for future research.

This study demonstrates that radiomics imaging biomarkers effectively identify NSCLC patients responsive to EGFR-TKIs, ICIs, and combination therapies. Further correlation analysis revealed a negative or non-significant association between PD-1/PD-L1 expression and EGFR wild-type status, in contrast to a significant positive correlation in EGFR-mutant NSCLC. These findings align with multiple preclinical studies [38, 39], indicating that NSCLC patients with EGFR mutation exhibit higher PD-L1 positivity rates than those with EGFR wild-type status. This phenomenon may be attributed to the role of intracellular EGFR signaling in directly or indirectly upregulating PD-L1 expression in tumor cells, thereby facilitating T-cell apoptosis and promoting immune evasion.

The observed modest correlation between PD-1/PD-L1 expression and the 19Del and L858R mutations suggests that these two common EGFR mutation subtypes may lead to EGFR activation and induce PD-L1 expression, as the downstream pathways of EGFR activation can be influenced by tumor suppressor genes and cytokines produced by the inflammatory microenvironment. In contrast, a negative or non-significant correlation was identified between the drug-resistant T790M mutation and PD-1/PD-L1 expression, suggesting that strong PD-L1 expression may be more prevalent in T790M-negative NSCLC patients than in their T790M-positive counterparts. These findings are consistent with the results reported by Inomata et al. [40].

These association results suggest that the response to EGFR-TKI and ICI therapies may not depend on a single tumor characteristic or a specific tumor cell type. However, it is important to note that the underlying explanations for these associations and their specific molecular mechanisms have not been fully elucidated, and further studies are required to gain a deeper understanding.

Another important observation from the findings is that the training set data should be heterogeneous. A large but overly homogeneous sample can impair the ability to discern responses to EGFR-TKIs and ICIs, as the derived algorithms require diverse training to identify patients with atypical characteristics effectively. For example, the radiomics-clinical model failed to identify two cases in an independent test set. One patient exhibited a 19Del mutation alongside a primary T790M mutation, while the other presented with a common L858R mutation in exon 21, along with MET amplification. The inability of the radiomics-clinical model to accurately categorize these cases may be attributed to the absence of these less common genetic alterations in the training set.

There are several limitations to our work. First, all study samples were retrospectively collected from different institutions, which may introduce selection bias during patient recruitment, potentially affecting the unbiased nature of the prediction model. Second, although radiomics analysis incorporating multi-region features enhances the robustness of the model, it is still limited by the manual extraction of radiomic features. Deep learning techniques are expected to improve medical image analysis further. Third, the radiomics imaging biomarkers identified in this study lacked an in-depth assessment of the underlying molecular mechanisms, which could be further explored by integrating genomics and proteomics in future studies. Fourth, the clinical utility of the developed predictive models in predicting treatment responses to EGFR-TKIs and ICIs requires further validation in larger and more heterogeneous prospective cohorts. Finally, novel automated segmentation algorithms need to be developed to simplify the radiomics process and better meet the demands of real-world clinical scenarios.

## Conclusion

In conclusion, this study demonstrates that radiomics-clinical model integrates multiregional features and clinical factors and is a non-invasive and efficient method for assessing the response of NSCLC patients to EGFR-TKIs and ICIs therapies. The model has potential in precision medicine and may become an important complementary tool for molecular biology analysis after further prospective validation. Moreover, the method’s potential is further enhanced by balancing protecting patient privacy and improving predictive accuracy. The developed imaging biomarkers for radiomics revealed potential associations between EGFR-TKIs therapy, ICI therapy, and their combination therapy. However, further molecular mechanism analysis still needs to validate and thoroughly investigate these findings.

## Electronic supplementary material

Below is the link to the electronic supplementary material.

Supplementary Material 1

## Data Availability

All resources associated with this study, including source code, data partitions, model parameter configurations, and scripts for model development and evaluation, are publicly accessible on GitHub: https://github.com/zxpShare/Radiomics-Clinical-NSCLC.

## Abbreviations

NSCLC: Non-small cell lung cancer
EGFR: Epidermal growth factor receptor
19Del: Exon 19 deletion
EGFR-TKIs: EGFR tyrosine kinase inhibitors
ICIs: Immune checkpoint inhibitors
PD-1: Programmed cell death protein 1
PD-L1: Programmed cell death ligand 1
AUC: Area under the curve
ROI: Region of interest
DCA: Decision curve analysis
RNA-seq: RNA sequencing
TPS: Tumor proportion score
FDR: False discovery rate
