# Supplementary material for: Evaluation of EGFR-TKIs and ICIs treatment stratification in non-small cell lung cancer using an encrypted multidimensional radiomics approach
Source: Cancer Imaging. 2025 Jan 20;25:3. doi: 10.1186/s40644-025-00824-w (PMC11748245; doi:10.1186/s40644-025-00824-w)
Supplement: Supplementary file 1 — Supplementary Material 1 [file 40644_2025_824_MOESM1_ESM.docx]

1. Recruitment criteria

We searched the medical record system of the First Affiliated Hospital of Gannan Medical University (China, Cohort I) to identify patients with imaging data. Inclusion criteria were as follows: (1) histologically confirmed NSCLC; (2) a chest CT scan obtained within one month before surgery or biopsy; (3) detection of EGFR mutation status, including 19Del, L858R, and T790M, or detection of PD-1/PD-L1 expression status; and (4) availability of complete clinicopathologic data. Exclusion criteria included: (1) antitumor therapy before the chest CT examination; (2) poor CT image quality for segmentation; and (3) patients with other malignancies.

Patients meeting the same criteria as Cohort I were selectively enrolled from the publicly accessible 'NSCLC Radiogenomics' dataset (USA, Cohort II), available on the Cancer Imaging Archive (TCIA). The download and use of their data adhered to the citation guidelines of the TCIA portal.

1. EGFR mutation and immunophenotype

EGFR mutation testing was conducted using real-time fluorescent polymerase chain reaction on tumor tissue samples obtained through biopsy. The genetic test report provided retrospective information on each patient's EGFR mutation subtype status, including 19Del, L858R, and T790M. If no EGFR exon 18-21 mutation was detected, the tumor was considered EGFR wild-type.

The PD-1/PD-L1 expression status was determined through immunohistochemical staining of formalin-fixed paraffin-embedded samples, utilizing the tumor proportion score (TPS) for categorization into expressed (TPS $\geq$ 1%) and unexpressed (TPS $<$ 1%) groups. Notably, the clinical data for Cohort II did not explicitly include information on patients' PD-1/PD-L1 expression status but provided associated RNA-seq data instead. Recent evidence supports a strong correlation between CD8 cell infiltration in the tumor microenvironment and the response to anti-PD-1/PD-L1 therapy [1,2]. To stratify patients, we used the median value of CD8 cell abundance estimated through the CD8B gene. We categorized them into groups with high and low tumor infiltration, construed as PD-1/PD-L1 expressed and unexpressed, respectively, for terminological consistency.

1. Parameters of the CT Scanners

Enrolled patients at the authors' institution underwent CT examinations using two scanning devices, but the procedures were similar. The parameters for the GE Revolution scanner (USA, 256-channel multi-detector) were as follows: tube energy, 100 kV; tube current, automated tube current modulation; rotation time, 0.5 s; detector collimation, 80x0.5 mm; arterial phase, 25–30 s after injection; venous phase, 60 s after injection. The parameters for the second scanner, a SOMATOM Definition scanner (Siemens Medical Systems, Germany, 64-channel multi-detector), were set as follows: tube energy, 100 kV; tube current, automated tube current modulation; rotation time, 0.5 s; detector collimation, 80x0.5 mm; arterial phase, 25–30 s after injection; venous phase, 60 s after injection. All preoperative images were retrieved from the Picture Archiving and Communication Systems (PACS) in the DICOM format.

1. Tumor segmentation

Tumor segmentation in 3D ROIs utilized 3D Slicer software (version 4.11). Initially, a respiratory physician with five years of expertise independently annotated tumor areas across CT slices, disregarding radiology reports. A second radiologist, possessing a decade of experience, subsequently reviewed and refined the ROIs, resolving disagreements through discussion. The annotation process meticulously considered the contours of the adjacent mediastinum, chest wall, and blood vessels, excluding bronchi, large vessels, veins, and normal tissue, whenever possible. In instances of multiple lesions, the primary tumor region was exclusively chosen to minimize clustering effects. An in-house algorithm identified slices with the largest tumor cross-sectional area (2D ROIs), and the 3D ROIs were then extended outward by 3 mm to create peritumoral ROIs. Except for the initial 3D ROIs segmentation for Cohort I (as corresponding 3D ROIs already existed for Cohort II), the subsequent 2D and peritumoral ROIs for both cohorts adhered to the same scheme.

1. Resampling analysis and design

Image resampling plays a crucial role in radiomic analysis as the reconstruction protocol for sections emphasizes clinical visual assessment over a detailed characterization of the tumor region. Voxel spacing inconsistencies, particularly with slice spacing exceeding in-plane spacing, add complexity exacerbated by variations in resolution from diverse imaging protocols and scanners. To address voxel anisotropy, all CT images in the study cohort undergo resampling into isotropic voxel space, ensuring a precise representation of spatial information. However, this introduces unavoidable distortion, requiring a delicate equilibrium between mitigating in-plane information loss from downsampling and addressing out-of-plane information introduced by upsampling. Careful consideration is essential when resampling 3D and peritumoral ROIs spanning multiple slices, in contrast to 2D ROIs disregarding slice thickness.

This study utilized the B-spline interpolation algorithm to resample all CT images, resulting in voxel dimensions of 0.8 mm × 0.8 mm for 2D ROIs and 1.0 mm × 1.0 mm × 1.0 mm for 3D and peritumoral ROIs. The determination of these voxel sizes was guided by examining the resolution distribution observed within the cohort across 2D, 3D, and peritumoral ROIs, focusing on prioritizing information fidelity.

The CT images, featuring slice thicknesses ranging from 0.6 mm to 5.0 mm, exhibited a prevalent voxel spacing pattern of either [0.686, 0.686, 0.7] mm or [0.75, 0.75, 0.7] mm. A strategic approach was adopted to leverage the benefits of different modalities, employing a medium voxel size (1.0 mm) for 3D and peritumoral modalities and a smaller voxel size (0.8 mm) for the 2D mode. This intentional voxel size selection aims to enhance the imaging data's analytical precision and sensitivity, tailored to each ROI category's specific requirements.

1. Advanced encryption standard (AES) algorithm
   1. *Encryption*

AES uses a block cipher structure with a block size of 128 bits and supports key lengths of 128, 192, or 256 bits, with the 256-bit key being the most secure. Data is processed in groups of four bytes called “states.” AES performs 10, 12, or 14 rounds, depending on the key length. During these rounds, the data undergoes four basic steps: SubBytes, ShiftRows, MixColumns, and AddRoundKey. The process is as follows:

AddRoundKey: In each round of AES, the input state is XORed with a 16-byte round key generated from the master key using a separate key expansion process, the only part that utilizes the user key. Therefore, this key is present only during the AddRoundKey phase. At other stages, both the beginning and the end can be reversed without knowing the key. Assuming that the byte in the ith row and jth column of the state matrix is $s_{i,j}$, and the byte in the ith row and jth column of the key matrix is $k_{i,j}$, the AddRoundKey operation is performed by executing $s_{i,j}=s_{i,j}\bigoplus k_{i,j}$​, under the conditions 1$\leq s_{i,j}\leq4$ and 1$\leq k_{i,j}\leq4$ [3].

SubBytes: This step uses a table called an S-box, a matrix of byte values. During SubBytes, each byte of the state matrix is replaced by the corresponding byte in the S-box. This replacement is done by taking the first 4 bits of each byte as the row value and the next 4 bits as the column value, then using these values to look up the new byte in the S-box, replacing the original byte in the state matrix. According to the AES specification, this S-box is fixed and is not generated or changed each time encryption is performed.

ShiftRows: In the ShiftRows step, all bytes in each row of the state matrix are cyclically shifted to the left, except for the first row, which remains unchanged. The second row is shifted 1 byte to the left, the third is shifted 2 bytes to the left, and the fourth is shifted 3 bytes to the left.

MixColumns: In the MixColumns step, each column of the state matrix is processed independently. Each byte in a column is remapped to a new value through a linear combination of all four bytes in that column. This step can be viewed as a reversible linear transformation, where each column is treated as a polynomial over the finite field GF ($2^{8}$) and multiplied by the fixed polynomial $c\left( x \right)=3x^{3}+x^{2}+x+2$ with modulus $x^{4}+1$ [3]. In other words, each column of the state matrix after the ShiftRows operation is multiplied by a fixed coefficient matrix. The specific matrix multiplication is expressed as follows:

$$\begin{aligned} \left[ \begin{matrix} \begin{matrix} 02 & 03 \\ 01 & 02 \end{matrix} & \begin{matrix} 01 & 01 \\ 03 & 01 \end{matrix} \\ \begin{matrix} 01 & 01 \\ 03 & 01 \end{matrix} & \begin{matrix} 02 & 03 \\ 01 & 02 \end{matrix} \end{matrix} \right]\left[ \begin{matrix} \begin{matrix} s_{0,0} & s_{0,1} \\ s_{1,0} & s_{1,1} \end{matrix} & \begin{matrix} s_{0,2} & s_{0,3} \\ s_{1,2} & s_{1,3} \end{matrix} \\ \begin{matrix} s_{2,0} & s_{2,1} \\ s_{3,0} & s_{3,1} \end{matrix} & \begin{matrix} s_{2,2} & s_{2,3} \\ s_{3,2} & s_{3,3} \end{matrix} \end{matrix} \right]=\left[ \begin{matrix} \begin{matrix} s_{0,0}^{'} & s_{0,1}^{'} \\ s_{1,0}^{'} & s_{1,1}^{'} \end{matrix} & \begin{matrix} s_{0,2}^{'} & s_{0,3}^{'} \\ s_{1,2}^{'} & s_{1,3}^{'} \end{matrix} \\ \begin{matrix} s_{2,0}^{'} & s_{2,1}^{'} \\ s_{3,0}^{'} & s_{3,1}^{'} \end{matrix} & \begin{matrix} s_{2,2}^{'} & s_{2,3}^{'} \\ s_{3,2}^{'} & s_{3,3}^{'} \end{matrix} \end{matrix} \right]\#(1) \end{aligned}$$

Combining the MixColumns transformation with the ShiftRows transformation ensures that all output bits depend on all input bits after several rounds of encryption. The first round of AES consists of four transformation steps: SubBytes, ShiftRows, MixColumns, and AddRoundKey. However, the MixColumns transformation is omitted in the last round, where only SubBytes, ShiftRows, and an additional AddRoundKey operation are performed. Additionally, an initial AddRoundKey transformation is applied before the first round, commonly called round 0.

In addition to the four steps mentioned above, AES includes a key expansion step utilized during the AddRoundKey process. The AES key expansion algorithm begins with an initial 128-bit key and expands it into a linear array of 44, 52, or 60 32-bit words, depending on the version of AES used. This results in a 4-word round key for the initial AddRoundKey phase and each round of encryption.

First, the 128-bit key is copied into the first four words of the extended key. The algorithm then cyclically populates the remaining parts of the extended key by adding 4-word blocks at a time. Each new 4-word block depends on the previous word and the first four-word blocks. Generally, new word blocks are generated using a simple XOR operation; however, the calculation becomes more complex every four indexes. A cyclic left shift is performed on the word block bytes, and then each byte is replaced using an S-box. Finally, the results of these steps are XORed with a round constant, and the resulting block is added to the extended key array.

- 1. Decryption

In the standard form, the AES decryption key is the same as the encryption key, as the transformation sequences (SubBytes, ShiftRows, MixColumns, AddRoundKey) in the decryption process are executed in reverse order. This means that encryption and decryption applications typically need two independent software implementations. However, it is possible to construct an equivalent decryption algorithm with the same structure and order as the encryption algorithm, except that the transformation functions are replaced with their corresponding inverse functions. Additionally, to ensure the equivalence of the two algorithms, the key scheduling must also be adjusted accordingly.

InvSubBytes: The inverse byte substitution function operates similarly to the SubBytes function but uses an inverse S-box. For example, 75 maps to 9D in the regular S-box, while 9D maps back to 75 in the inverse S-box.

InvShiftRows: The reverse row shift transformation operates like the ShiftRows function, but the shift direction is reversed; the rows are shifted to the right instead of to the left.

InvMixColumns: The inverse column mixing transform operation is similar to the MixColumns function, but it uses the inverse matrix of the one used by MixColumns. Specifically, the matrix (2) is replaced with its inverse matrix (3).

$$\begin{aligned} \left[ \begin{matrix} \begin{matrix} 02 & 03 \\ 01 & 02 \end{matrix} & \begin{matrix} 01 & 01 \\ 03 & 01 \end{matrix} \\ \begin{matrix} 01 & 01 \\ 03 & 01 \end{matrix} & \begin{matrix} 02 & 03 \\ 01 & 02 \end{matrix} \end{matrix} \right] \left( 2 \right) \left[ \begin{matrix} \begin{matrix} 0E & 0B \\ 09 & 0E \end{matrix} & \begin{matrix} 0D & 09 \\ 0B & 0D \end{matrix} \\ \begin{matrix} 0D & 09 \\ 0B & 0D \end{matrix} & \begin{matrix} 0E & 0B \\ 09 & 0E \end{matrix} \end{matrix} \right]\#\left（ 3 \right） \end{aligned}$$

InvAddRoundKey: The inverse round key transformation is the same as the AddRoundKey function because the XOR operation is self-inverse. For example, $A \bigoplus B\oplus B=A$.

- 1. Encrypted blockchain

Special care must be taken when selecting the block cipher mode of AES for encrypting medical images due to the high correlation and repetitiveness of pixel values in chest CT images. Modes like electronic codebooks are unsuitable, as repeated plaintext blocks yield identical ciphertext blocks, enabling an attacker to deduce the original information. Instead, cipher-block chaining (CBC) is a more suitable option.

Even if a plaintext block is reused in CBC mode, it will generate a different ciphertext block. This is because the encryption input consists of the XOR result of the current plaintext block and the previous ciphertext block. If the last block is incomplete, this mode requires filling it in using a secure padding function.

- 1. Key generation

After anonymizing the data, the patient's medical record number is selected as the key variable. The medical record number is unique and can be accurately linked to a single patient visit's electronic medical record data. Next, a password-based key derivation function (PBKDF2) generates the key. This method enhances the computational complexity of key generation through multiple iterations and adds a salt value, thereby improving key security. Different salt values result in the generation of different keys. Even if an unauthorized person obtains the medical record number, using a salt value can effectively mitigate the risk of key disclosure.

1. Machine learning classifiers

We employed seven commonly used machine learning classifiers to identify suitable classification algorithms for various scenarios and enhance the robustness of the study results. These include logistic regression, linear discriminant analysis, random forest, k-nearest neighbors, Naive Bayes, support vector machine, and multi-layer perceptron. Previous studies have validated the hypothesis that these classifiers demonstrate good predictive power in determining gene mutation status or the expression level of immune molecules in tumor patients.

1. RadScore subgroups

This study determined optimal RadScore threshold values for predicting four EGFR mutation states and one immunophenotype based on the RadScore quartiles in the training dataset. For EGFR mutant and wild-type, patients were categorized into the RadScore-High group if RadScore was >= 0.0948 and the RadScore-Low group if RadScore < -0.090. Those with RadScore >= -0.090 but < 0.0948 were placed in the RadScore-Median group.

Similarly, specific thresholds for other EGFR mutant subtypes were identified. For 19Del, the thresholds were -0.154 and 0.154 (RadScore-Low group: below -0.154, RadScore-High group: at or above 0.154, RadScore-Median group: [-0.154, 0.154]); for L858R, the thresholds were -0.129 and 0.177 (RadScore-Low group: below -0.129, RadScore-High group: at or above 0.177, RadScore-Median group: [-0.129, 0.177]); and for T790M, the thresholds were -0.158 and 0.212 (RadScore-Low group: below -0.158, RadScore-High group: at or above 0.212, RadScore-Median group: [-0.158, 0.212]).

Furthermore, in the context of PD-1/PD-L1 expression status subgroups, patients in the RadScore-High group were characterized by a RadScore greater than or equal to 0.093, those in the RadScore-Low group had a RadScore less than 0.098, and individuals in the RadScore-Median group exhibited RadScore values greater than or equal to -0.098 but less than 0.093.

1. Feature selection and model building

Among the EGFR mutation, a rigorous feature selection process revealed 10, 7, and 8 distinctive features within the training set corresponding to the 2D, 3D, and peritumoral regions, respectively. Following the identification of these features, RadScore (2D), RadScore (3D), and RadScore (peritumoral) were generated for each patient. Subsequently, radiomics models for the 2D, 3D, and peritumoral regions were created based on these RadScores.

Considering the added value of integrating clinical factors with the characteristics of different tumor regions, we initially established a foundational clinical model. Subsequently, we derived a RadScore (combined) using the same methodology applied to individual regions. This composite score integrated two features from 2D regions, four from 3D regions, and four from the peritumoral region. The significant clinical features and the RadScore (combined) were then utilized to construct the radiomics-clinical model.

In the context of EGFR mutant subtypes, we determined the optimal number of features derived from the 2D region, 3D region, peritumoral region, and the combined region as follows: 9, 6, 9, and 10 for the 19Del mutation; 9, 9, 8, and 9 for the L858R mutation; and 7, 9, 10, and 8 for the T790M mutation. RadScores for four distinct regions in each scenario were calculated using these selected features. From these RadScores and pertinent clinical features, we formulated five predictive models in each scenario using a congruent methodology.

In evaluating PD-1/PD-L1 expression, we employed ten features from the 2D region, ten from the 3D region, and eight from the peritumoral region. The resultant RadScores, derived from these distinctive features, were incorporated into the respective 2D, 3D, and peritumoral models. The RadScore (combined) was calculated after this integration, unifying two features from the 2D region, four from the 3D region, and four from the peritumoral region. Subsequently, we constructed clinical and radiomics-clinical models based on important clinical features and the RadScore (combined).

Appendix I.1 – I.5 summarizes the detailed formulas for RadScore (2D), RadScore (3D), RadScore (peritumoral), and RadScore (combined) in each of these groups.

- 1. *EGFR mutation status*

The formula for RadScores is as follows:

- RadScore (2D) = f_2D_1 *_ c_2D_1 +_ f_2D_2 *_ c_2D_2 +_ f_2D_3 *_ c_2D_3 +_ f_2D_4 *_ c_2D_4 +_ f_2D_5 *_ c_2D_5 +_ f_2D_6 *_ c_2D_6 +_ f_2D_7 *_ c_2D_7 +_ f_2D_8 *_ c_2D_8 +_ f_2D_9 *_ c_2D_9 +_ f_2D_10 *_ c_2D_10_
- RadScore (3D) = f_3D_1 *_ c_3D_1 +_ f_3D_2 *_ c_3D_2 +_ f_3D_3 *_ c_3D_3 +_ f_3D_4 *_ c_3D_4 +_ f_3D_5 *_ c_3D_5 +_ f_3D_6 *_ c_3D_6 +_ f_3D_7 *_ c_3D_7_
- RadScore (peritumoral) = f_ Per_1 *_ c_ Per_1 +_ f_ Per_2 *_ c_ Per_2 +_ f_ Per_3 *_ c_ Per_3 +_ f_ Per_4 *_ c_ Per_4 +_ f_ Per_5 *_ c_ Per_5 +_ f_ Per_6 *_ c_ Per_6 +_ f_ Per_7 *_ c_ Per_7 +_ f_ Per_8 *_ c_ Per_8_
- RadScore (combined) = f_ Com_1 *_ c_ Com_1 +_ f_ Com_2 *_ c_ Com_2 +_ f_ Com_3 *_ c_ Com_3 +_ f_ Com_4 *_ c_ Com_4 +_ f_ Com_5 *_ c_ Com_5 +_ f_ Com_6 *_ c_ Com_6 +_ f_ Com_7 *_ c_ Com_7 +_ f_ Com_8 *_ c_ Com_8 +_ f_ Com_9 *_ c_ Com_9 +_ f_ Com_10 *_ c_ Com_10_

where the abbreviations in the formula are shown in Table S2.

- 1. *19Del mutation status*

The formula for RadScores is as follows:

- RadScore (2D) = f_2D_1 *_ c_2D_1 +_ f_2D_2 *_ c_2D_2 +_ f_2D_3 *_ c_2D_3 +_ f_2D_4 *_ c_2D_4 +_ f_2D_5 *_ c_2D_5 +_ f_2D_6 *_ c_2D_6 +_ f_2D_7 *_ c_2D_7 +_ f_2D_8 *_ c_2D_8 +_ f_2D_9 *_ c_2D_9_
- RadScore (3D) = f_3D_1 *_ c_3D_1 +_ f_3D_2 *_ c_3D_2 +_ f_3D_3 *_ c_3D_3 +_ f_3D_4 *_ c_3D_4 +_ f_3D_5 *_ c_3D_5 +_ f_3D_6 *_ c_3D_6_
- RadScore (peritumoral) = f_ Per_1 *_ c_ Per_1 +_ f_ Per_2 *_ c_ Per_2 +_ f_ Per_3 *_ c_ Per_3 +_ f_ Per_4 *_ c_ Per_4 +_ f_ Per_5 *_ c_ Per_5 +_ f_ Per_6 *_ c_ Per_6 +_ f_ Per_7 *_ c_ Per_7 +_ f_ Per_8 *_ c_ Per_8 +_ f_ Per_9 *_ c_ Per_9_
- RadScore (combined) = f_ Com_1 *_ c_ Com_1 +_ f_ Com_2 *_ c_ Com_2 +_ f_ Com_3 *_ c_ Com_3 +_ f_ Com_4 *_ c_ Com_4 +_ f_ Com_5 *_ c_ Com_5 +_ f_ Com_6 *_ c_ Com_6 +_ f_ Com_7 *_ c_ Com_7 +_ f_ Com_8 *_ c_ Com_8 +_ f_ Com_9 *_ c_ Com_9 +_ f_ Com_10 *_ c_ Com_10_

where the abbreviations in the formula are shown in Table S3.

- 1. *L858R mutation status*

The formula for RadScores is as follows:

- RadScore (2D) = f_2D_1 *_ c_2D_1 +_ f_2D_2 *_ c_2D_2 +_ f_2D_3 *_ c_2D_3 +_ f_2D_4 *_ c_2D_4 +_ f_2D_5 *_ c_2D_5 +_ f_2D_6 *_ c_2D_6 +_ f_2D_7 *_ c_2D_7 +_ f_2D_8 *_ c_2D_8 +_ f_2D_9 *_ c_2D_9_
- RadScore (3D) = f_3D_1 *_ c_3D_1 +_ f_3D_2 *_ c_3D_2 +_ f_3D_3 *_ c_3D_3 +_ f_3D_4 *_ c_3D_4 +_ f_3D_5 *_ c_3D_5 +_ f_3D_6 *_ c_3D_6 +_ f_3D_7 *_ c_3D_7 +_ f_3D_8 *_ c_3D_8 +_ f_3D_9 *_ c_3D_9_
- RadScore (peritumoral) = f_ Per_1 *_ c_ Per_1 +_ f_ Per _2 *_ c_ Per_2 +_ f_ Per_3 *_ c_ Per_3 +_ f_ Per_4 *_ c_ Per_4 +_ f_ Per_5 *_ c_ Per_5 +_ f_ Per_6 *_ c_ Per_6 +_ f_ Per_7 *_ c_ Per_7 +_ f_ Per_8 *_ c_ Per_8_
- RadScore (combined) = f_ Com_1 *_ c_ Com_1 +_ f_ Com_2 *_ c_ Com_2 +_ f_ Com_3 *_ c_ Com_3 +_ f_ Com_4 *_ c_ Com_4 +_ f_ Com_5 *_ c_ Com_5 +_ f_ Com_6 *_ c_ Com_6 +_ f_ Com_7 *_ c_ Com_7 +_ f_ Com_8 *_ c_ Com_8 +_ f_ Com_9 *_ c_ Com_9_

where the abbreviations in the formula are shown in Table S4.

- 1. *T790M mutation status*

The formula for RadScores is as follows:

- RadScore (2D) = f_2D_1 *_ c_2D_1 +_ f_2D_2 *_ c_2D_2 +_ f_2D_3 *_ c_2D_3 +_ f_2D_4 *_ c_2D_4 +_ f_2D_5 *_ c_2D_5 +_ f_2D_6 *_ c_2D_6 +_ f_2D_7 *_ c_2D_7_
- RadScore (3D) = f_3D_1 *_ c_3D_1 +_ f_3D_2 *_ c_3D_2 +_ f_3D_3 *_ c_3D_3 +_ f_3D_4 *_ c_3D_4 +_ f_3D_5 *_ c_3D_5 +_ f_3D_6 *_ c_3D_6 +_ f_3D_7 *_ c_3D_7 +_ f_3D_8 *_ c_3D_8 +_ f_3D_9 *_ c_3D_9_
- RadScore (peritumoral) = f_ Per_1 *_ c_ Per_1 +_ f_ Per_2 *_ c_ Per_2 +_ f_ Per_3 *_ c_ Per_3 +_ f_ Per_4 *_ c_ Per_4 +_ f_ Per_5 *_ c_ Per_5 +_ f_ Per_6 *_ c_ Per_6 +_ f_ Per_7 *_ c_ Per_7 +_ f_ Per_8 *_ c_ Per_8 +_ f_ Per_9 *_ c_ Per_9 +_ f_ Per_10 *_ c_ Per_10_
- RadScore (combined) = f_ Com_1 *_ c_ Com_1 +_ f_ Com_2 *_ c_ Com_2 +_ f_ Com_3 *_ c_ Com_3 +_ f_ Com_4 *_ c_ Com_4 +_ f_ Com_5 *_ c_ Com_5 +_ f_ Com_6 *_ c_ Com_6 +_ f_ Com_7 *_ c_ Com_7 +_ f_ Com_8 *_ c_ Com_8_

where the abbreviations in the formula are shown in Table S5.

- 1. *PD-1/PD-L1 expression levels*

The formula for RadScores is as follows:

- RadScore (2D) = f_2D_1 *_ c_2D_1 +_ f_2D_2 *_ c_2D_2 +_ f_2D_3 *_ c_2D_3 +_ f_2D_4 *_ c_2D_4 +_ f_2D_5 *_ c_2D_5 +_ f_2D_6 *_ c_2D_6 +_ f_2D_7 *_ c_2D_7 +_ f_2D_8 *_ c_2D_8 +_ f_2D_9 *_ c_2D_9 +_ f_2D_10 *_ c_2D_10_
- RadScore (3D) = f_3D_1 *_ c_3D_1 +_ f_3D_2 *_ c_3D_2 +_ f_3D_3 *_ c_3D_3 +_ f_3D_4 *_ c_3D_4 +_ f_3D_5 *_ c_3D_5 +_ f_3D_6 *_ c_3D_6 +_ f_3D_7 *_ c_3D_7 +_ f_3D_8 *_ c_3D_8 +_ f_3D_9 *_ c_3D_9 +_ f_3D_10 *_ c_3D_10_
- RadScore (peritumoral) = f_ Per_1 *_ c_ Per_1 +_ f_ Per_2 *_ c_ Per_2 +_ f_ Per_3 *_ c_ Per_3 +_ f_ Per_4 *_ c_ Per_4 +_ f_ Per_5 *_ c_ Per_5 +_ f_ Per_6 *_ c_ Per_6 +_ f_ Per_7 *_ c_ Per_7 +_ f_ Per_8 *_ c_ Per_8_
- RadScore (combined) = f_ Com_1 *_ c_ Com_1 +_ f_ Com_2 *_ c_ Com_2 +_ f_ Com_3 *_ c_ Com_3 +_ f_ Com_4 *_ c_ Com_4 +_ f_ Com_5 *_ c_ Com_5 +_ f_ Com_6 *_ c_ Com_6 +_ f_ Com_7 *_ c_ Com_7 +_ f_ Com_8 *_ c_ Com_8 +_ f_ Com_9 *_ c_ Com_9 +_ f_ Com_10 *_ c_ Com_10_

where the abbreviations in the formula are shown in Table S6.

Tables

Table S1. The codebase and parameter settings utilized in feature extraction.

| Code Libraries | Parameter Settings |
| --- | --- |
| PyRadiomics | |
| imageType | Original |
| featureClass | shape, firstorder, glcm, glrlm, glszm, gldm, ngtdm |
| interpolator | sitkBSpline |
| binWidth | 25 |
| voxelArrayShift | 1000 |
| resampledPixelSpacing | determined by experiment |

Table S2. The best features selected and their respective RadScores calculation formulas for predicting EGFR mutation.

|  | Radiomic feature | Abbreviation | Coefficient | Abbreviation |
| --- | --- | --- | --- | --- |
| 2D ROI | | | | |
|  | wavelet-LLL_glrlm_HighGrayLevelRunEmphasis | f_2D_1_ | -0.108871675 | c_2D_1_ |
|  | wavelet-HHL_glrlm_RunLengthNonUniformityNormalized | f_2D_2_ | -0.147745792 | c_2D_2_ |
|  | original_firstorder_Range | f_2D_3_ | 0.107229971 | c_2D_3_ |
|  | wavelet-LLL_glrlm_RunLengthNonUniformity | f_2D_4_ | -0.049887848 | c_2D_4_ |
|  | wavelet-HHH_gldm_HighGrayLevelEmphasis | f_2D_5_ | 0.044710173 | c_2D_5_ |
|  | wavelet-LLH_glcm_Correlation | f_2D_6_ | 0.050499192 | c_2D_6_ |
|  | wavelet-HLL_glcm_MCC | f_2D_7_ | 0.065041307 | c_2D_7_ |
|  | wavelet-LLH_glrlm_LongRunHighGrayLevelEmphasis | f_2D_8_ | -0.040441318 | c_2D_8_ |
|  | wavelet-HLH_firstorder_Mean | f_2D_9_ | 0.072565236 | c_2D_9_ |
|  | wavelet-HHL_firstorder_Median | f_2D_10_ | -0.053691055 | c_2D_10_ |
| 3D ROI | | | | |
|  | wavelet-HHL_gldm_HighGrayLevelEmphasis | f_3D_1_ | -0.050096415 | c_3D_1_ |
|  | wavelet-HHL_glcm_Imc1 | f_3D_2_ | 0.02105312 | c_3D_2_ |
|  | wavelet-HLH_glszm_SizeZoneNonUniformity | f_3D_3_ | -0.118191624 | c_3D_3_ |
|  | original_glcm_Correlation | f_3D_4_ | 0.056796937 | c_3D_4_ |
|  | wavelet-HLH_firstorder_Skewness | f_3D_5_ | -0.036277513 | c_3D_5_ |
|  | wavelet-LLL_glcm_Imc1 | f_3D_6_ | 0.082142755 | c_3D_6_ |
|  | original_shape_Flatness | f_3D_7_ | -0.042282807 | c_3D_7_ |
| Peritumoral ROI | | | | |
|  | wavelet-HHH_firstorder_90Percentile | f_Per_1_ | -0.081936899 | c_Per_1_ |
|  | original_firstorder_MeanAbsoluteDeviation | f_Per_2_ | -0.027300706 | c_Per_2_ |
|  | original_shape_SurfaceArea | f_Per_3_ | -0.037067788 | c_Per_3_ |
|  | wavelet-HHH_firstorder_Skewness | f_Per_4_ | -0.08574615 | c_Per_4_ |
|  | wavelet-LHL_firstorder_Median | f_Per_5_ | 0.062583091 | c_Per_5_ |
|  | wavelet-HLL_glcm_MCC | f_Per_6_ | -0.009155088 | c_Per_6_ |
|  | wavelet-LHL_glcm_Imc2 | f_Per_7_ | -0.035159759 | c_Per_7_ |
|  | wavelet-HLL_glszm_GrayLevelVariance | f_Per_8_ | -0.002458381 | c_Per_8_ |
| Combined | | | | |
|  | wavelet-HHH_firstorder_90Percentile_peritumoral | f_Com_1_ | -0.018807022 | c_Com_1_ |
|  | wavelet-HLH_glszm_SizeZoneNonUniformity_3D | f_Com_2_ | -0.070342272 | c_Com_2_ |
|  | wavelet-HHH_firstorder_Skewness_peritumoral | f_Com_3_ | -0.054886128 | c_Com_3_ |
|  | wavelet-LLL_glrlm_HighGrayLevelRunEmphasis_2D | f_Com_4_ | -0.026243881 | c_Com_4_ |
|  | original_shape_Flatness_3D | f_Com_5_ | -0.042239835 | c_Com_5_ |
|  | wavelet-HLH_firstorder_Mean_2D | f_Com_6_ | -0.027464672 | c_Com_6_ |
|  | wavelet-LHL_glcm_Imc2_peritumoral | f_Com_7_ | -0.052624772 | c_Com_7_ |
|  | original_firstorder_MeanAbsoluteDeviation_peritumoral | f_Com_8_ | -0.023742854 | c_Com_8_ |
|  | original_glcm_Correlation_3D | f_Com_9_ | 0.07427312 | c_Com_9_ |
|  | wavelet-HHL_glcm_Imc1_3D | f_Per_10_ | 0.039944477 | c_Com_10_ |

Table S3. The best features selected and their respective RadScores calculation formulas for predicting 19Del mutation.

|  | Radiomic feature | Abbreviation | Coefficient | Abbreviation |
| --- | --- | --- | --- | --- |
| 2D ROI | | | | |
|  | wavelet-LHH_gldm_HighGrayLevelEmphasis | f_2D_1_ | -0.049236626 | c_2D_1_ |
|  | original_glcm_ClusterProminence | f_2D_2_ | -0.149523421 | c_2D_2_ |
|  | original_shape_SurfaceVolumeRatio | f_2D_3_ | 0.035416681 | c_2D_3_ |
|  | original_gldm_LargeDependenceHighGrayLevelEmphasis | f_2D_4_ | 0.066933353 | c_2D_4_ |
|  | wavelet-LHH_glszm_LargeAreaEmphasis | f_2D_5_ | 0.004276323 | c_2D_5_ |
|  | wavelet-HHH_glszm_GrayLevelVariance | f_2D_6_ | -0.076451859 | c_2D_6_ |
|  | wavelet-LHL_firstorder_Maximum | f_2D_7_ | 0.050837042 | c_2D_7_ |
|  | wavelet-HHH_firstorder_Median | f_2D_8_ | -0.034796111 | c_2D_8_ |
|  | wavelet-LLH_firstorder_Skewness | f_2D_9_ | 0.100995364 | c_2D_9_ |
| 3D ROI | | | | |
|  | wavelet-HHH_glszm_ZoneVariance | f_3D_1_ | 0.048235067 | c_3D_1_ |
|  | wavelet-LLH_firstorder_Range | f_3D_2_ | -0.099073175 | c_3D_2_ |
|  | wavelet-LLH_glcm_Correlation | f_3D_3_ | -0.160208506 | c_3D_3_ |
|  | wavelet-HLL_firstorder_Skewness | f_3D_4_ | 0.175033834 | c_3D_4_ |
|  | wavelet-HHL_firstorder_RobustMeanAbsoluteDeviation | f_3D_5_ | -0.172428066 | c_3D_5_ |
|  | wavelet-HHH_firstorder_Mean | f_3D_6_ | -0.033587067 | c_3D_6_ |
| Peritumoral ROI | | | | |
|  | wavelet-HHH_glrlm_RunVariance | f_Per_1_ | 0.078816023 | c_Per_1_ |
|  | wavelet-HHH_glcm_SumAverage | f_Per_2_ | 0.052347718 | c_Per_2_ |
|  | wavelet-LLL_glszm_LargeAreaLowGrayLevelEmphasis | f_Per_3_ | -0.110730764 | c_Per_3_ |
|  | wavelet-HHL_glcm_Autocorrelation | f_Per_4_ | 0.01034568 | c_Per_4_ |
|  | wavelet-LLL_firstorder_Energy | f_Per_5_ | -0.115871756 | c_Per_5_ |
|  | wavelet-HLL_glcm_Imc2 | f_Per_6_ | -0.135855609 | c_Per_6_ |
|  | original_gldm_SmallDependenceHighGrayLevelEmphasis | f_Per_7_ | -0.181059032 | c_Per_7_ |
|  | wavelet-HLL_firstorder_Minimum | f_Per_8_ | 0.028431642 | c_Per_8_ |
|  | original_firstorder_Maximum | f_Per_9_ | 0.080416791 | c_Per_9_ |
| Combined | | | | |
|  | wavelet-HHH_glrlm_RunVariance_peritumoral | f_Com_1_ | 0.146554606 | c_Com_1_ |
|  | original_glcm_ClusterProminence_2D | f_Com_2_ | -0.042582047 | c_Com_2_ |
|  | wavelet-HLL_glcm_Imc2_peritumoral | f_Com_3_ | -0.085861254 | c_Com_3_ |
|  | wavelet-HLL_firstorder_Skewness_3D | f_Com_4_ | 0.135991641 | c_Com_4_ |
|  | wavelet-LLH_glcm_Correlation_3D | f_Com_5_ | -0.071693495 | c_Com_5_ |
|  | wavelet-HHH_firstorder_Mean_3D | f_Com_6_ | -0.041890485 | c_Com_6_ |
|  | original_firstorder_Maximum_peritumoral | f_Com_7_ | 0.037431854 | c_Com_7_ |
|  | wavelet-LLL_firstorder_Energy_peritumoral | f_Com_8_ | -0.039982602 | c_Com_8_ |
|  | wavelet-HHH_glszm_GrayLevelVariance_2D | f_Com_9_ | -0.020553329 | c_Com_9_ |
|  | wavelet-LLH_firstorder_Skewness_2D | f_Per_10_ | 0.03905543 | c_Com_10_ |

Table S4. The best features selected and their respective RadScores calculation formulas for predicting L858R mutation.

|  | Radiomic feature | Abbreviation | Coefficient | Abbreviation |
| --- | --- | --- | --- | --- |
| 2D ROI | | | | |
|  | wavelet-LLL_gldm_SmallDependenceHighGrayLevelEmphasis | f_2D_1_ | -0.018586068 | c_2D_1_ |
|  | wavelet-HHH_glszm_ZoneEntropy | f_2D_2_ | -0.148524976 | c_2D_2_ |
|  | wavelet-HHL_glszm_GrayLevelNonUniformity | f_2D_3_ | -0.093464015 | c_2D_3_ |
|  | wavelet-HHH_glszm_LowGrayLevelZoneEmphasis | f_2D_4_ | -0.104001133 | c_2D_4_ |
|  | wavelet-HLH_glcm_JointAverage | f_2D_5_ | -0.070431595 | c_2D_5_ |
|  | wavelet-HHL_glcm_MCC | f_2D_6_ | -0.046395909 | c_2D_6_ |
|  | original_shape_Elongation | f_2D_7_ | -0.074072309 | c_2D_7_ |
|  | wavelet-LHH_gldm_DependenceVariance | f_2D_8_ | 0.02390011 | c_2D_8_ |
|  | wavelet-LHL_glcm_MCC | f_2D_9_ | -0.058007603 | c_2D_9_ |
| 3D ROI | | | | |
|  | wavelet-LLL_glcm_ClusterProminence | f_3D_1_ | -0.078160712 | c_3D_1_ |
|  | wavelet-HLH_glcm_SumAverage | f_3D_2_ | 0.253866983 | c_3D_2_ |
|  | original_glszm_SizeZoneNonUniformityNormalized | f_3D_3_ | -0.054011102 | c_3D_3_ |
|  | wavelet-HHL_glcm_Autocorrelation | f_3D_4_ | -0.048764121 | c_3D_4_ |
|  | wavelet-HHH_glszm_SizeZoneNonUniformity | f_3D_5_ | -0.052588874 | c_3D_5_ |
|  | original_glszm_LargeAreaLowGrayLevelEmphasis | f_3D_6_ | -0.1126671 | c_3D_6_ |
|  | wavelet-LHH_glcm_Autocorrelation | f_3D_7_ | -0.279623404 | c_3D_7_ |
|  | original_shape_Flatness | f_3D_8_ | -0.086579216 | c_3D_8_ |
|  | wavelet-HLH_glszm_SizeZoneNonUniformityNormalized | f_3D_9_ | -0.042476493 | c_3D_9_ |
| Peritumoral ROI | | | | |
|  | wavelet-LHH_gldm_HighGrayLevelEmphasis | f_Per_1_ | -0.001276208 | c_Per_1_ |
|  | wavelet-HLL_glcm_ClusterShade | f_Per_2_ | 0.164790068 | c_Per_2_ |
|  | wavelet-HHL_gldm_HighGrayLevelEmphasis | f_Per_3_ | -0.184951478 | c_Per_3_ |
|  | wavelet-HLH_glcm_JointAverage | f_Per_4_ | 0.114971799 | c_Per_4_ |
|  | wavelet-LHL_glrlm_GrayLevelNonUniformityNormalized | f_Per_5_ | 0.002618596 | c_Per_5_ |
|  | wavelet-LLL_firstorder_Minimum | f_Per_6_ | 0.138800345 | c_Per_6_ |
|  | wavelet-LHH_glszm_LargeAreaEmphasis | f_Per_7_ | 0.348400597 | c_Per_7_ |
|  | wavelet-HHH_glcm_ClusterShade | f_Per_8_ | -0.039221581 | c_Per_8_ |
| Combined | | | | |
|  | wavelet-LLL_glcm_ClusterProminence_3D | f_Com_1_ | -0.03626722 | c_Com_1_ |
|  | wavelet-LHH_gldm_DependenceVariance_2D | f_Com_2_ | 0.031226418 | c_Com_2_ |
|  | wavelet-HLH_glcm_JointAverage_2D | f_Com_3_ | -0.074926426 | c_Com_3_ |
|  | wavelet-HLH_glcm_JointAverage_peritumoral | f_Com_4_ | 0.143365085 | c_Com_4_ |
|  | wavelet-LLL_gldm_SmallDependenceHighGrayLevelEmphasis_2D | f_Com_5_ | -0.034508133 | c_Com_5_ |
|  | wavelet-HHL_glszm_GrayLevelNonUniformity_2D | f_Com_6_ | -0.080793807 | c_Com_6_ |
|  | original_shape_Elongation_2D | f_Com_7_ | -0.06491815 | c_Com_7_ |
|  | wavelet-LLL_firstorder_Minimum_peritumoral | f_Com_8_ | 0.197989853 | c_Com_8_ |
|  | wavelet-LHH_gldm_HighGrayLevelEmphasis_peritumoral | f_Com_9_ | 0.028762683 | c_Com_9_ |

Table S5. The best features selected and their respective RadScores calculation formulas for predicting T790M mutation.

|  | Radiomic feature | Abbreviation | Coefficient | Abbreviation |
| --- | --- | --- | --- | --- |
| 2D ROI | | | | |
|  | original_gldm_DependenceVariance | f_2D_1_ | -0.031002023 | c_2D_1_ |
|  | wavelet-HHL_glcm_Autocorrelation | f_2D_2_ | -0.091747854 | c_2D_2_ |
|  | wavelet-HHH_glszm_GrayLevelNonUniformity | f_2D_3_ | -0.053869027 | c_2D_3_ |
|  | wavelet-HHL_glszm_LargeAreaLowGrayLevelEmphasis | f_2D_4_ | 0.045337869 | c_2D_4_ |
|  | wavelet-HHH_glszm_GrayLevelNonUniformityNormalized | f_2D_5_ | 0.033906022 | c_2D_5_ |
|  | wavelet-HLL_firstorder_TotalEnergy | f_2D_6_ | -0.031113542 | c_2D_6_ |
|  | original_shape_Elongation | f_2D_7_ | 0.105318854 | c_2D_7_ |
| 3D ROI | | | | |
|  | wavelet-HHL_glcm_Autocorrelation | f_3D_1_ | 0.112418906 | c_3D_1_ |
|  | wavelet-HLH_ngtdm_Contrast | f_3D_2_ | -0.00584973 | c_3D_2_ |
|  | original_firstorder_Maximum | f_3D_3_ | -0.16343868 | c_3D_3_ |
|  | original_firstorder_Range | f_3D_4_ | -0.181978364 | c_3D_4_ |
|  | wavelet-LHL_glszm_LargeAreaLowGrayLevelEmphasis | f_3D_5_ | -0.164828293 | c_3D_5_ |
|  | wavelet-LLL_firstorder_Mean | f_3D_6_ | 0.157848463 | c_3D_6_ |
|  | wavelet-HLH_glcm_JointAverage | f_3D_7_ | -0.031778652 | c_3D_7_ |
|  | wavelet-HLH_glszm_SizeZoneNonUniformity | f_3D_8_ | 0.089465594 | c_3D_8_ |
|  | original_gldm_LargeDependenceHighGrayLevelEmphasis | f_3D_9_ | 0.048427663 | c_3D_9_ |
| Peritumoral ROI | | | | |
|  | wavelet-LLH_glszm_SmallAreaHighGrayLevelEmphasis | f_Per_1_ | -0.071442531 | c_Per_1_ |
|  | wavelet-HHH_gldm_HighGrayLevelEmphasis | f_Per_2_ | -0.023210905 | c_Per_2_ |
|  | original_glszm_LargeAreaLowGrayLevelEmphasis | f_Per_3_ | -0.053836611 | c_Per_3_ |
|  | wavelet-HLL_glcm_Idmn | f_Per_4_ | -0.049904244 | c_Per_4_ |
|  | wavelet-LHH_glcm_JointAverage | f_Per_5_ | -0.004871466 | c_Per_5_ |
|  | wavelet-LLL_glcm_SumEntropy | f_Per_6_ | 0.122209219 | c_Per_6_ |
|  | wavelet-HLH_glrlm_HighGrayLevelRunEmphasis | f_Per_7_ | -0.079345468 | c_Per_7_ |
|  | wavelet-HHH_glszm_ZoneVariance | f_Per_8_ | -0.017576598 | c_Per_8_ |
|  | original_firstorder_InterquartileRange | f_Per_9_ | 0.089015455 | c_Per_9_ |
|  | original_gldm_DependenceNonUniformity | f_Per_10_ | -0.151729132 | c_Per_10_ |
| Combined | | | | |
|  | wavelet-HLH_ngtdm_Contrast_3D | f_Com_1_ | 0.024503004 | c_Com_1_ |
|  | original_firstorder_Maximum_3D | f_Com_2_ | -0.100869853 | c_Com_2_ |
|  | wavelet-HLH_glcm_JointAverage_3D | f_Com_3_ | -0.111345537 | c_Com_3_ |
|  | original_gldm_DependenceVariance_2D | f_Com_4_ | 0.04161976 | c_Com_4_ |
|  | original_glszm_LargeAreaLowGrayLevelEmphasis_peritumoral | f_Com_5_ | -0.058631718 | c_Com_5_ |
|  | wavelet-HHL_glszm_LargeAreaLowGrayLevelEmphasis_2D | f_Com_6_ | -0.004132575 | c_Com_6_ |
|  | wavelet-LLL_glcm_SumEntropy_peritumoral | f_Com_7_ | 0.13632873 | c_Com_7_ |
|  | original_firstorder_InterquartileRange_peritumoral | f_Com_8_ | 0.083568868 | c_Com_8_ |

Table S6. The best features selected and their respective RadScores calculation formulas for predicting PD-1/PD-L1 expression.

|  | Radiomic feature | Abbreviation | Coefficient | Abbreviation |
| --- | --- | --- | --- | --- |
| 2D ROI | | | | |
|  | wavelet-LLH_ngtdm_Contrast | f_2D_1_ | 0.095000796 | c_2D_1_ |
|  | wavelet-HLL_glrlm_LongRunHighGrayLevelEmphasis | f_2D_2_ | -0.081702605 | c_2D_2_ |
|  | original_shape_Elongation | f_2D_3_ | 0.065863899 | c_2D_3_ |
|  | original_glrlm_LowGrayLevelRunEmphasis | f_2D_4_ | -0.147709469 | c_2D_4_ |
|  | wavelet-HLL_glcm_MCC | f_2D_5_ | -0.001066606 | c_2D_5_ |
|  | original_glcm_MCC | f_2D_6_ | 0.131110539 | c_2D_6_ |
|  | wavelet-HLH_ngtdm_Strength | f_2D_7_ | -0.047320536 | c_2D_7_ |
|  | wavelet-LLH_firstorder_TotalEnergy | f_2D_8_ | -0.201709586 | c_2D_8_ |
|  | wavelet-LHL_glcm_Imc1 | f_2D_9_ | 0.248420026 | c_2D_9_ |
|  | wavelet-LHL_gldm_SmallDependenceLowGrayLevelEmphasis | f_2D_10_ | 0.009774009 | c_2D_10_ |
| 3D ROI | | | | |
|  | original_shape_Elongation | f_3D_1_ | -0.046762858 | c_3D_1_ |
|  | original_shape_Sphericity | f_3D_2_ | -0.085362613 | c_3D_2_ |
|  | wavelet-HHL_glcm_Imc1 | f_3D_3_ | -0.142494975 | c_3D_3_ |
|  | wavelet-HHH_firstorder_Mean | f_3D_4_ | -0.183139924 | c_3D_4_ |
|  | wavelet-LHL_firstorder_Skewness | f_3D_5_ | -0.045923993 | c_3D_5_ |
|  | wavelet-HLL_glcm_MCC | f_3D_6_ | 0.057452265 | c_3D_6_ |
|  | wavelet-HLL_firstorder_Skewness | f_3D_7_ | 0.060207969 | c_3D_7_ |
|  | wavelet-LLL_gldm_LargeDependenceLowGrayLevelEmphasis | f_3D_8_ | -0.084091144 | c_3D_8_ |
|  | wavelet-LLH_glcm_Imc2 | f_3D_9_ | -0.075485457 | c_3D_9_ |
|  | wavelet-HLH_firstorder_Skewness | f_3D_10_ | -0.115483124 | c_3D_10_ |
| Peritumoral ROI | | | | |
|  | original_firstorder_MeanAbsoluteDeviation | f_Per_1_ | -0.002388638 | c_Per_1_ |
|  | wavelet-HLH_firstorder_Skewness | f_Per_2_ | -0.149418845 | c_Per_2_ |
|  | wavelet-HLL_firstorder_Median | f_Per_3_ | 0.134973625 | c_Per_3_ |
|  | wavelet-LHL_glcm_MCC | f_Per_4_ | -0.006378277 | c_Per_4_ |
|  | wavelet-HHL_firstorder_Median | f_Per_5_ | 0.143830443 | c_Per_5_ |
|  | wavelet-LHH_firstorder_Skewness | f_Per_6_ | 0.122212606 | c_Per_6_ |
|  | wavelet-LHL_ngtdm_Complexity | f_Per_7_ | -0.058242249 | c_Per_7_ |
|  | original_firstorder_Energy | f_Per_8_ | -0.043806471 | c_Per_8_ |
| Combined | | | | |
|  | wavelet-LLH_ngtdm_Contrast_2D | f_Com_1_ | -0.020342647 | c_Com_1_ |
|  | original_shape_Elongation_3D | f_Com_2_ | 0.057570896 | c_Com_2_ |
|  | wavelet-LLH_glcm_ClusterShade_3D | f_Com_3_ | 0.079789868 | c_Com_3_ |
|  | wavelet-HLH_firstorder_Skewness_3D | f_Com_4_ | -0.010095781 | c_Com_4_ |
|  | original_firstorder_Energy_peritumoral | f_Com_5_ | -0.013035417 | c_Com_5_ |
|  | wavelet-HLL_glrlm_LongRunLowGrayLevelEmphasis_3D | f_Com_6_ | -0.024495216 | c_Com_6_ |
|  | wavelet-HHL_firstorder_Median_peritumoral | f_Com_7_ | 0.091294118 | c_Com_7_ |
|  | wavelet-LLH_firstorder_Mean_2D | f_Com_8_ | -0.084985542 | c_Com_8_ |
|  | wavelet-LHH_firstorder_Skewness_peritumoral | f_Com_9_ | 0.021881819 | c_Com_9_ |
|  | wavelet-LHL_glcm_MCC_peritumoral | f_Com_10_ | -0.078292139 | c_Com_10_ |

Table S7. Correlation coefficient between adjacent pixels in original and encrypted images.

| Patients | Original image | | |  | Encrypted image | | |
| --- | --- | --- | --- | --- | --- | --- | --- |
|  | Horizontal direction | Vertical direction | Diagonal direction |  | Horizontal direction | Vertical direction | Diagonal direction |
| 1 | 0.9822 | 0.9873 | 0.9780 |  | -0.0039 | -0.0028 | -0.0015 |
| 2 | 0.9902 | 0.9918 | 0.9865 |  | 0.0012 | -0.0033 | 0.0019 |
| 3 | 0.9748 | 0.9822 | 0.9697 |  | 0.0020 | -0.0012 | -0.0017 |
| 4 | 0.9817 | 0.9900 | 0.9749 |  | -0.0017 | -0.0025 | 0.0029 |
| 5 | 0.9903 | 0.9914 | 0.9864 |  | 0.0009 | 0.0022 | 0.0008 |
| 6 | 0.9905 | 0.9921 | 0.9875 |  | -0.0013 | 0.0015 | -0.0006 |

Table S8. Predictive performance of five models in the independent test dataset.

|  | Model | Classifier | Accuracy | Precision | Recall | F1 Score | AUC (95% CI) |
| --- | --- | --- | --- | --- | --- | --- | --- |
| EGFR mutation | Clinical | LDA | 0.691 | 0.679 | 0.685 | 0.685 | 0.708 (0.697, 0.718) |
|  | 2D radiomics | SVM | 0.693 | 0.731 | 0.729 | 0.693 | 0.730 (0.720, 0.740) |
|  | 3D radiomics | LR | 0.723 | 0.722 | 0.721 | 0.721 | 0.746 (0.737, 0.755) |
|  | Peritumoral radiomics | SVM | 0.746 | 0.730 | 0.731 | 0.730 | 0.752 (0.742, 0.761) |
|  | Radiomics-Clinical | NB | 0.839 | 0.828 | 0.836 | 0.831 | 0.884 (0.877, 0.890) |
| 19Del mutation | Clinical | NB | 0.710 | 0.672 | 0.691 | 0.677 | 0.715 (0.701, 0.728) |
|  | 2D radiomics | RF | 0.721 | 0.706 | 0.769 | 0.707 | 0.756 (0.741, 0.772) |
|  | 3D radiomics | NB | 0.731 | 0.731 | 0.805 | 0.724 | 0.777 (0.767, 0.787) |
|  | Peritumoral radiomics | LR | 0.713 | 0.680 | 0.693 | 0.704 | 0.734 (0.718, 0.749) |
|  | Radiomics-Clinical | LDA | 0.838 | 0.807 | 0.868 | 0.806 | 0.894 (0.887, 0.901) |
| L858R mutation | Clinical | NB | 0.707 | 0.611 | 0.685 | 0.611 | 0.728 (0.713, 0.745) |
|  | 2D radiomics | NB | 0.726 | 0.681 | 0.746 | 0.706 | 0.753 (0.739, 0.767) |
|  | 3D radiomics | NB | 0.767 | 0.712 | 0.757 | 0.755 | 0.784 (0.772, 0.797) |
|  | Peritumoral radiomics | SVM | 0.686 | 0.665 | 0.740 | 0.693 | 0.746 (0.729, 0.763) |
|  | Radiomics-Clinical | LDA | 0.860 | 0.824 | 0.849 | 0.834 | 0.881 (0.867, 0.896) |
| T790M mutation | Clinical | MLP | 0.712 | 0.667 | 0.713 | 0.644 | 0.746 (0.719, 0.774) |
|  | 2D radiomics | KNN | 0.768 | 0.747 | 0.684 | 0.700 | 0.769 (0.750, 0.786) |
|  | 3D radiomics | SVM | 0.792 | 0.736 | 0.751 | 0.802 | 0.816 (0.796, 0.835) |
|  | Peritumoral radiomics | RF | 0.733 | 0.693 | 0.770 | 0.699 | 0.771 (0.757, 0.784) |
|  | Radiomics-Clinical | LR | 0.876 | 0.831 | 0.826 | 0.845 | 0.900 (0.889, 0.910) |
| PD-1/PD-L1 expression | Clinical | MLP | 0.670 | 0.727 | 0.661 | 0.642 | 0.716 (0.700, 0.732) |
|  | 2D radiomics | SVM | 0.766 | 0.815 | 0.759 | 0.754 | 0.754 (0.737, 0.769) |
|  | 3D radiomics | RF | 0.762 | 0.773 | 0.766 | 0.760 | 0.774 (0.758, 0.789) |
|  | Peritumoral radiomics | LR | 0.765 | 0.767 | 0.766 | 0.765 | 0.779 (0.764, 0.792) |
|  | Radiomics-Clinical | LDA | 0.842 | 0.859 | 0.849 | 0.848 | 0.893 (0.883, 0.903) |

Table S9. Comparison of the efficiency between the AES-CBC algorithm and radiomics feature extraction for all patients.

| Type | Library | Resampling | **Decryption (s)** | Feature extraction (s) | Total (s) |
| --- | --- | --- | --- | --- | --- |
| 2D ROI | Pyradiomics | $0.8mm \times0.8mm$ | **1.93 (15.37%)** | 10.62 (84.63%) | 12.55 |
| 3D ROI |  | $10mm\times1.0mm1.0mm$ | **2.00 (14.36%)** | 11.93 (85.64%) | 13.93 |
| Peritumoral ROI |  | $10mm\times1.0mm1.0mm$ | **2.09 (12.99%)** | 14.00 (87.01%) | 16.09 |
| Note: Data are presented as the mean time (in seconds) unless otherwise indicated. Abbreviations: ROI, region of interest. | | | | | |

References

1. Sun R, Limkin E J, Vakalopoulou M, et al. A radiomics approach to assess tumour-infiltrating CD8 cells and response to anti-PD-1 or anti-PD-L1 immunotherapy: an imaging biomarker, retrospective multicohort study[J]. The Lancet Oncology, 2018, 19(9): 1180-1191.
2. Wu J, Li C, Gensheimer M, et al. Radiological tumour classification across imaging modality and histology[J]. Nature machine intelligence, 2021, 3(9): 787-798.
3. Subramani S, Svn S K. Review of security methods based on classical cryptography and quantum cryptography[J]. Cybernetics and Systems, 2023: 1-19.
